# *Caesalpinia mimosoides* Leaf Extract Promotes Neurite Outgrowth and Inhibits BACE1 Activity in Mutant APP-Overexpressing Neuronal Neuro2a Cells

**DOI:** 10.3390/ph14090901

**Published:** 2021-09-04

**Authors:** Panthakarn Rangsinth, Chatrawee Duangjan, Chanin Sillapachaiyaporn, Ciro Isidoro, Anchalee Prasansuklab, Tewin Tencomnao

**Affiliations:** 1 Graduate Program in Clinical Biochemistry and Molecular Medicine, Department of Clinical Chemistry, Faculty of Allied Health Sciences, Chulalongkorn University, Bangkok 10330, Thailand; panthakarn.rangsinth@gmail.com (P.R.); chatrawee.d1992@gmail.com (C.D.); chanin.sill@gmail.com (C.S.); 2 Department of Health Sciences, Università del Piemonte Orientale “A. Avogadro”, Via Solaroli 17, 28100 Novara, Italy; ciro.isidoro@med.uniupo.it; 3 Natural Products for Neuroprotection and Anti-Ageing Research Unit, Chulalongkorn University, Bangkok 10330, Thailand; 4 College of Public Health Sciences, Chulalongkorn University, Bangkok 10330, Thailand; 5 Department of Clinical Chemistry, Faculty of Allied Health Sciences, Chulalongkorn University, Bangkok 10330, Thailand

**Keywords:** Alzheimer’s disease, neurodegenerative diseases, Neuro2a/APPSwe, neuritogenesis, amyloid precursor protein, BACE1, molecular docking, ADMET analysis

## Abstract

Alzheimer’s disease (AD) is implicated in the imbalance of several proteins, including Amyloid-β (Aβ), amyloid precursor protein (APP), and BACE1. APP overexpression interferes with neurite outgrowth, while BACE1 plays a role in Aβ generation. Medicinal herbs with effects on neurite outgrowth stimulation and BACE1 inhibition may benefit AD. This study aimed to investigate the neurite outgrowth stimulatory effect, along with BACE1 inhibition of *Caesalpinia mimosoides* (CM), using wild-type (Neuro2a) and APP (Swedish mutant)-overexpressing (Neuro2a/APPSwe) neurons. The methanol extract of CM leaves stimulated neurite outgrowth in wild-type and APP-overexpressing cells. After exposure to the extract, the mRNA expression of the neurite outgrowth activation genes growth-associated protein-43 (GAP-43) and teneurin-4 (Ten-4) was increased in both Neuro2a and Neuro2a/APPSwe cells, while the mRNA expression of neurite outgrowth negative regulators Nogo receptor (NgR) and Lingo-1 was reduced. Additionally, the extract suppressed BACE1 activity in the APP-overexpressing neurons. Virtual screening demonstrated that quercetin-3′-glucuronide, quercetin-3-O-glucoside, clausarinol, and theogallin were possible inhibitors of BACE1. ADMET was analyzed to predict drug-likeness properties of CM-constituents. These results suggest that CM extract promotes neurite outgrowth and inhibits BACE1 activity in APP-overexpressing neurons. Thus, CM may serve as a source of drugs for AD treatment. Additional studies for full identification of bioactive constituents and to confirm the neuritogenesis in vivo are needed for translation into clinic of the present findings.

## 1. Introduction

Alzheimer’s disease (AD) is a progressive neurodegenerative disease and the most common form of dementia among elder people; it seriously affects a person’s memory and ability to carry out daily activities. An effective treatment for AD is still not available. AD is characterized by loss of neurons and synapses in the brain, particularly in the hippocampus area. The cause of this disease is not fully understood. It is believed that the disease process is associated with the accumulation of amyloid-β (Aβ) peptide generated by the cleavage of amyloid precursor protein (APP) by beta-secretase (BACE-1) in the amyloidogenic pathway [1,2].

The disease process is associated with hyperphosphorylated tau protein, a microtubule assembly protein accumulating intracellularly as neurofibrillary tangles (NFTs) and Aβ peptide deposited in diffuse and neuritic plaques in the brain [3]. Both tangles and plaques are found in the brains of individuals afflicted by AD [4,5]. It is still unclear what are the causes leading to abnormal hyperphosphorylation of tau and whether Aβ accumulation occurs due to its overproduction or a defect in the clearance [6,7,8].

Aβ, a hallmark protein found in patients with AD, arises from a protein called amyloid precursor protein (APP) that is processed via β- and γ-secretase cleavage [9]. The mutation in APP gene also influences Aβ level in the brain. The well-known Swedish mutation in APP has been reported to increase Aβ production and secretion [10,11]. Moreover, overexpression of APP has been reported to inhibit cell differentiation and neurite outgrowth in cultured Neuro2a cells [12]. Therefore, the therapeutic intervention of AD by inducing neuroregeneration or neurite outgrowth should also consider the condition of APP overexpression.

The reconstruction of the neuronal and synaptic networks for the recovery of brain functions is a potential therapeutic strategy for AD. One of the neuro-regeneration processes is neuritogenesis or neurite outgrowth, which is a branching of neurites followed by elongation of axons and dendrites in maturing neuron [13]. This process is an important step to construct the functional networks of neurons and is considered a hallmark of neuronal differentiation [14]. Previous findings have shown that overexpression and mutation of APP have an inhibitory influence on neurite outgrowth [12,15]. Moreover, recent study showed that overexpressing APP with the Swedish mutation results in a physical interaction between APP and the negative neurite outgrowth regulator Lingo-1 [16].

Leucine rich repeat and immunoglobin-like domain-containing protein 1 (Lingo-1) is a transmembrane protein highly expressed in the brain. Lingo-1 has been implicated in several neurodegenerative diseases, including AD [17,18]. Its action remarkably relates to the Nogo receptor (NgR) as part of a co-receptor complex leading to activation of rho-associated coiled coil-containing protein kinase (RhoA/ROCK) signaling pathway, which subsequently suppresses the development of growth cones and axon [19,20].

Typically, neurite outgrowth can be induced by several neurotrophic factors, including nerve growth factor (NGF) and brain-derived neurotrophic factor (BDNF) [21,22]. Unfortunately, NGF level is found to decrease during aging [23,24,25]. Moreover, NGF is a large polypeptide that cannot pass the blood–brain barrier [26,27], so the treatment using NGF must be given directly to the brain [28]. Hence, searching for novel small molecules with neurite outgrowth promoting activity may constitute an alternative therapy for AD. Importantly, these molecules should also exert the effect even in neurons with APP overexpression, the condition mimicking AD pathology.

In recent years, medicinal plant-derived natural compounds have received extensive attention as major sources of new therapeutic agents for treating neurodegenerative diseases and neurological disorders. Many of them have been shown to exert their neurotrophic effects by promoting neurite outgrowth [29,30,31].

*Caesalpinia mimosoides* Lam., a small spiny and woody climbing tropical trees belonging to Fabaceae family and Caesalpinioideae subfamily, is native to Southeast Asia and to Northern and Northeastern parts of Thailand. Young twigs and leaves are edible and are traditionally used as an anti-flatulent and a remedy against fainting and dizziness [32]. The plant has been reported to exhibit antioxidant [32,33], anti-inflammatory [34], anti-cancer [35], and anti-aging activities [33]. Moreover, *C. mimosoides* contains several bioactive compounds, including gallic acid [35] and quercetin [36], that have been previously reported to have neurite outgrowth activity [37,38,39]. Recently, quercetin isolated from *C. mimosoides* was shown to possess neurite outgrowth and neuroprotective properties against acetylcholinesterase (AChE) in cultured P19-derived neurons [36]. However, the neurite outgrowth stimulatory and BACE1 inhibitory effect of this plant on neuronal cells overexpressing APP has not been investigated.

In this study, the effect of APP overexpression on neurite outgrowth was investigated, along with the reversing effect of *C. mimosoided* (CM) extract against APP-overexpressing neuronal cells. Swedish mutant APP-overexpressing Neuro2a (Neuro2a/APPSwe) cells were employed for comparison with Neuro2a cells expressing wild-type APP [40,41]. The mechanisms underlying the activity of CM extract were examined by studying the expression of several signaling molecules involved in neurite outgrowth. Moreover, the effect of CM extract on BACE1 inhibition was investigated in the cells along with in silico approaches. Furthermore, drug-likeliness, bioavailability, and toxicity of the CM-constituents were predicted using ADMET analysis.

## 2. Results

### 2.1. Quantification of Gallic Acid and Quercetin

**Figure 1:**
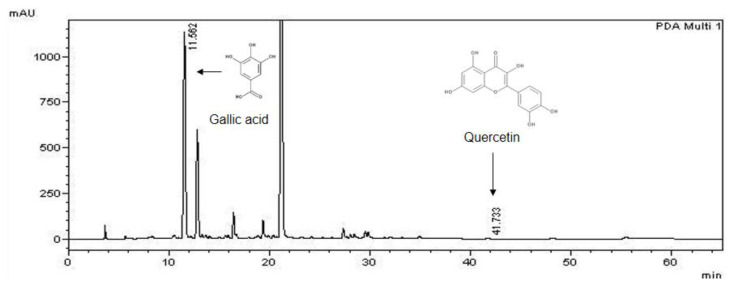
HPLC chromatogram. Gallic acid and quercetin in CM methanol extract were quantified using HPLC analysis. The peaks at the retention time of 11.56 and 41.73 min represented as gallic acid and quercetin, respectively.

Previously, we have reported the phytochemical profiling by LC-MS of CM methanol extract used in the current study [33]. The major compounds in the extract included gallic acid and quercetin, which were shown to possess neurite outgrowth activity [36,37,38,39]. Hence, the amount of these compounds was quantified. The HPLC chromatogram of CM methanol extract showed peaks representing gallic acid and quercetin at the retention time of 11.56 and 41.73 min, respectively (Figure 1). Based on the calculations of the external standard, the methanol extract contained gallic acid and quercetin at 7815.17 ± 25.09 mg and 36.33 ± 0.51 mg of compounds per 100 g crude extract, respectively.

### 2.2. Selection of CM Extract Concentration

**Figure 2:**
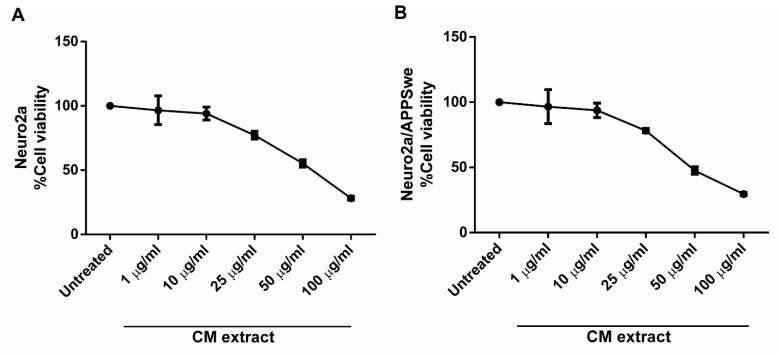
MTT assay. Cell viability of (**A**) Neuro2a and (**B**) Neuro2a/APPSwe cells after treatment with the various concentrations of CM extract for 48 h. The extract concentration of 10 µg/mL with more than 90% cell survival was chosen as the test concentration for subsequent experiments.

To select the optimal concentration of CM methanol extract, an MTT assay was employed to assess cellular toxicity of the extract at a series of different concentration. After treatment of Neuro2a and Neuro2a/APPSwe cells with the various concentrations (1, 10, 25, 50, and 100 µg/mL) of CM extract for 48 h, a concentration-dependent toxicity effect in both neuronal cell lines with more than 50% reduction of viable cells at the high concentrations tested (25, 50 and 100 µg/mL) was observed. The maximum concentration of the extract that produced acceptable toxicity with a minimum cell viability of 90% in both Neuro2a and Neuro2a/APPSwe cells was found at 10 µg/mL (94.08 ± 4.98% and 93.78 ± 5.56%, respectively) (Figure 2A,B). According to these results, 10 µg/mL of CM methanol extract was, therefore, selected for subsequent experiments.

### 2.3. Effects of CM Extract on Neurite Outgrowth Activity

**Figure 3:**
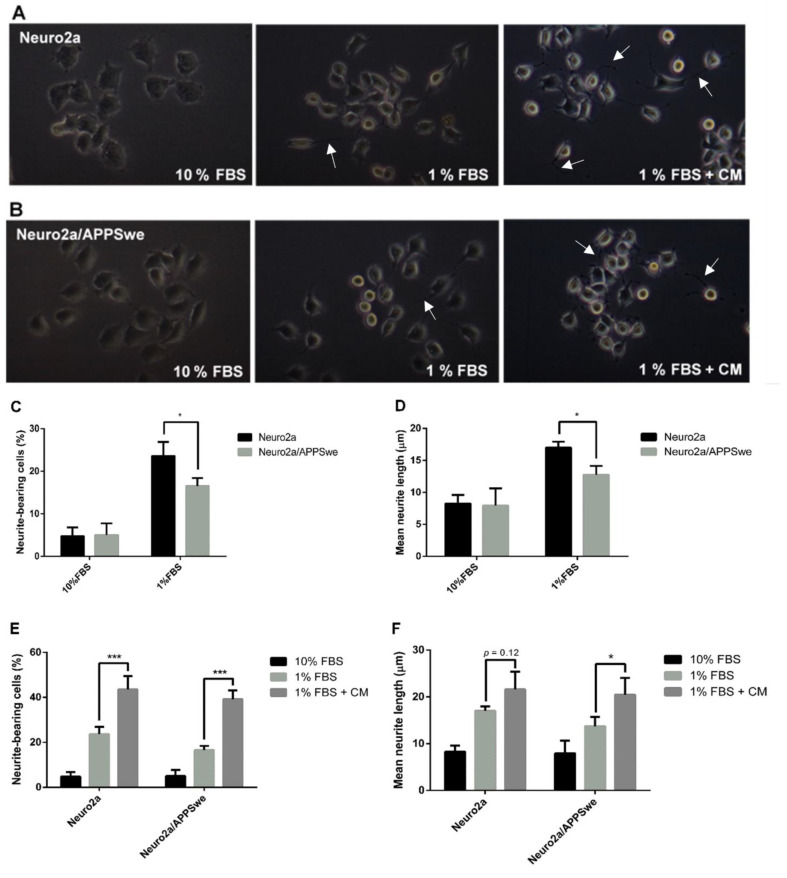
Neurite outgrowth determination. The neuronal morphology of (**A**) Neuro2a cells. (**B**) Neuro2a/APPSwe cells. Neuro2a/APPSwe cells exhibited number of (**C**) neurite-bearing cells and (**D**) neurite length significantly lower than Neuro2a cells after induced differentiation by 1% FBS medium for 48 h. When treated with 10 µg/mL CM extract for 48 h, Neuro2a and Neuro2a/APPSwe cells significantly increased both number of (**E**) neurite-bearing cells and (**F**) neurite length when compared with 1% FBS treatment. The white arrow indicates neurite. Values are mean ± SD of at least three independent experiments. * *p*-value < 0.05, *** *p*-value < 0.001.

Serum deprivation is a condition for inducing neurite outgrowth in Neuro2a cells [42]. Therefore, we investigated neurite outgrowth activity of Neuro2a and Neuro2a/APPSwe cells after treatment in 1% FBS differentiation medium (with or without CM extract). The complete medium containing 10% FBS was used as an undifferentiated control. Neuronal morphology after treatment with the different conditions for 48 h is shown in Figure 3A,B. We first investigated the effect of APP overexpression on neurite outgrowth activity. Neuro2a and Neuro2a/APPSwe cells were cultured in differentiation medium containing 1% FBS or complete medium containing 10% FBS (control) for 48 h. After culturing the cells in 1% FBS medium, the neurite outgrowth of Neuro2a and Neuro2a/APPSwe cells was increased in both cell types as percent of neurite-bearing cells (23.62 ± 3.27% and 16.56 ± 1.87%, respectively) and as the mean of the longest neurite length (17.01 ± 0.92 µm and 13.73 ± 1.97 µm, respectively) compared to the cells cultured in 10% FBS medium. When the comparison was done between cell lines, both percent of neurite-bearing cells and the mean of longest neurite length after treatment of differentiation medium were found to be significantly lower in Neuro2a/APPSwe cells than in Neuro2a cells (*p*-value = 0.03 and 0.02, respectively) (Figure 3C,D).

To investigate the ability to potentiate neurite outgrowth of CM extract, the cells were treated with the extract at a concentration of 10 µg/mL CM extract diluted in 1% FBS medium for 48 h, while cells receiving 10% FBS or 1% FBS medium treatment were used as undifferentiated and differentiated controls, respectively. The result showed that CM extract, when compared with 1% FBS medium control, significantly increased the percent of neurite-bearing cells (43.52 ± 6.00% vs. 23.62 ± 3.27%, *p*-value = 0.0025) and slightly (but not significantly) increased the mean neurite length (21.59 ± 3.76 µm vs. 17.01 ± 0.9 µm, *p*-value = 0.12) in Neuro2a cells. Meanwhile, Neuro2a/APPSwe cells treated with CM extract showed a significant increase in both percent of neurite-bearing cells (39.19 ± 3.87% vs. 16.56 ± 1.87%, *p*-value = 0.0025) and the mean neurite length (20.47 ± 3.56 µm vs. 13.73 ± 1.97 µm, *p*-value = 0.045) when compared with 1% FBS medium control (Figure 3E,F).

### 2.4. APP Expression in Neuro2a and Neuro2a/APPSwe Cells

**Figure 4:**
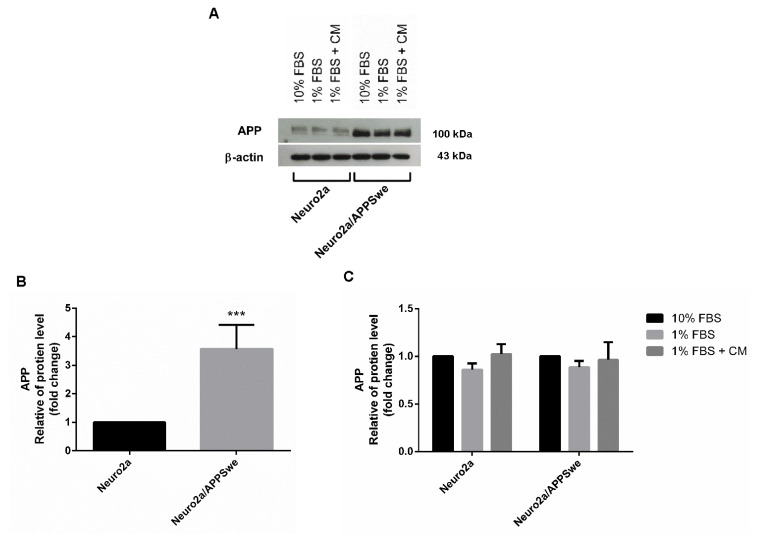
APP expression level. (**A**) Western blot analysis revealed that APP expression in Neuro2a/APPSwe cells was higher than that in Neuro2a cells. (**B**) In 1% FBS medium, APP was significantly different between wild-type cells and APPSwe-overexpressing Neuro2a cells (**C**) The APP levels between treatment groups of Neuro2a and Neuro2a/APPSwe cells were normalized vs. the 10% FBS group for each cell type. The analysis showed that Neuro2a/APPSwe cells exhibited higher levels of APP than Neuro2a cells. APP level in both cells did not differ in 10% FBS, 1% FBS, and 10 µg/mL CM extract in 1% FBS treatment. Values are mean ± SD of at least three independent experiments. *** *p*-value < 0.001.

Compared to Neuro2a cells, NeuroAPPSwe cells express higher levels of APP-bearing the Swedish mutation. Western blot analysis was used to determine APP level using antibodies specific to both wild-type and Swedish mutant APP. The protein bands showed that the level of APP was clearly different between wild-type cells and APP-overexpressing Neuro2a cells (Figure 4A,B). However, APP level was not altered after treatment with 10 µg/mL CM extract for 48 h in both cells (Figure 4C).

### 2.5. Effects of CM Extract on GAP-43 Gene Expression

**Figure 5:**
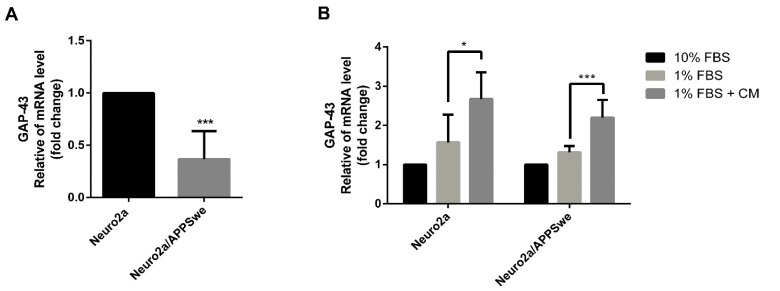
GAP-43 mRNA expression level. (**A**) Neuro2a/APPSwe cells showed significant lower GAP-43 gene expression than Neuro2a cells after induced differentiation by 1% FBS medium for 48 h. (**B**) The GAP-43 mRNA levels between treatment groups of Neuro2a and Neuro2a/APPSwe cells were normalized vs. 10% FBS group for each cell type. Neuro2a and Neuro2a/APPSwe cells significantly increased the expression of GAP-43 gene when treated with 10 µg/mL of CM extract for 48 h compared to 1% FBS treatment. Values are mean ± SD of at least three independent experiments. * *p*-value < 0.05, *** *p*-value < 0.001.

To further examine the mechanism underlying neurite outgrowth activity of CM methanol extract, gene expression of the neurite outgrowth marker, GAP-43, was investigated using quantitative real-time RT-PCR. By comparing both cell lines cultured in 1% FBS for 48 h, we found that the level of GAP-43 mRNA expression was significantly lower in the APP Swedish-mutant (Neuro2a/APPSwe) cells than in Neuro2a cells (*p*-value = 0.0002) (Figure 5A). After inducing cell differentiation in 1% FBS medium, GAP-43 mRNA level was increased in both Neuro2a and Neuro2a/APPSwe cells when compared with 10% FBS control. Worthy of note, in the presence of 10 µg/mL CM extract, both Neuro2a and Neuro2a/APPSwe cells showed a significant increase of GAP-43 expression level when compared to the cells in 1% FBS medium control (*p*-value = 0.046 and 0.0008, respectively) (Figure 5B).

### 2.6. Effects of and CM Extract on Ten-4 Gene Expression

**Figure 6:**
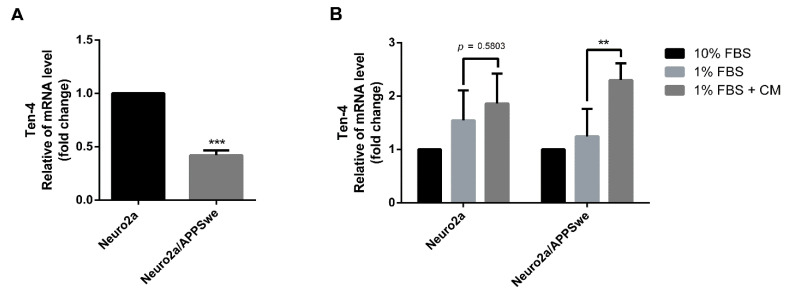
Ten-4 mRNA expression level. (**A**) Neuro2a/APPSwe cells showed significant lower Ten-4 gene expression than Neuro2a cells in 1% FBS medium after 48 h treatment. (**B**) The relative Ten-4 mRNA levels between treatment groups of Neuro2a and Neuro2a/APPSwe cell groups were normalized by 10% FBS group from each cell type. Ten-4 gene expression significantly increased in both Neuro2a and Neuro2a/APPSwe cells when treated with 10 µg/mL of CM extract for 48 h compared to 1% FBS treatment in each cell. Values are mean ± SD of at least three independent experiments. ** *p*-value < 0.01, *** *p*-value < 0.001.

The neuritogenesis signaling pathway of neurite outgrowth activity affected by APP overexpression and CM extract treatment was further examined. We evaluated the mRNA expression level of Teneurin-4 (Ten-4), which plays a key role in neuronal development and neurite outgrowth [43]. Following treatment of the cells with 1% FBS medium control for 48 h, Ten-4 mRNA expression was significantly lower in APP-overexpressing cells than in normal Neuro2a cells (*p*-value < 0.0001) (Figure 6A). Treating the cells with 1% FBS medium control along with 10 µg/mL CM extract significantly increased the expression level of Ten-4 mRNA in Neuro2a/APPSwe cells as compared to 1% FBS medium control (*p*-value = 0.007). In Neuro2a cells treated with 1% FBS medium plus CM extract, the mRNA level of Ten-4 also tended to increase when compared to 1% FBS medium control alone (*p*-value = 0.58) (Figure 6B). However, in comparison to 10% FBS medium control, Ten-4 mRNA expression level after the extract treatment was significantly higher in both Neuro2a cells (by 1.86-fold) and Neuro2a/APPSwe cells (by 2.3-fold). Notably, there was a slight increase of expression level for Ten-4 in APP overexpressing cells treated with 1% FBS medium control compared to 10% FBS medium control.

### 2.7. Effects of CM Extract on Lingo-1 Gene Expression

**Figure 7:**
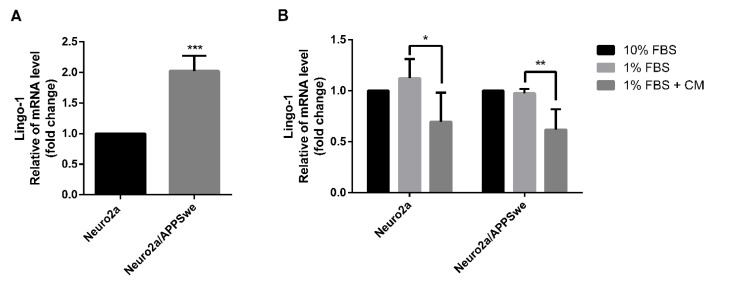
Lingo-1 mRNA expression level. (**A**) Neuro2a/APPSwe cells showed significant higher Lingo-1 gene expression level than Neuro2a cells in 1% FBS medium after 48 h treatment. (**B**) Lingo-1 mRNA level between treatment groups of Neuro2a and Neuro2a/APPSwe cells was normalized vs. 10% FBS group for each cell type. Lingo-1 gene expression level was significantly increased in both Neuro2a and Neuro2a/APPSwe cells when treated with 10 µg/mL of CM extract for 48 h compared to 1% FBS treatment in each cell. Values are mean ± SD of at least three independent experiments. * *p*-value < 0.05, ** *p*-value < 0.01, *** *p*-value < 0.001.

Lingo-1, also known as leucine rich repeat and immunoglobin-like domain-containing protein 1, is a transmembrane signaling protein that negatively regulates neurite outgrowth [20] and is shown to physically interact with APP [16]. By comparing mRNA expression level of Lingo-1 under differentiated condition induced by 1% FBS medium control, Swedish mutant APP-overexpressing neurons exhibited a significant higher gene expression than the wild-type neurons by 2.02-fold (*p*-value = 0.0002) (Figure 7A). The Lingo-1 mRNA level showed no significant difference between Neuro2a and Neuro2a/APPSwe cells when treated with 10% FBS or 1% FBS medium controls, but it significantly decreased in both cell lines after 48 h exposure to 1% FBS medium control along with 10 µg/mL CM extract when compared to 1% FBS medium control alone (*p*-value = 0.0372 and 0.0086, respectively) (Figure 7B).

### 2.8. Effects of CM Extract on NgR Gene Expression

**Figure 8:**
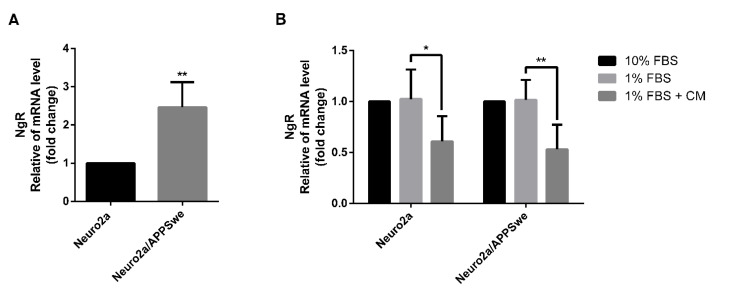
NgR mRNA expression level. (**A**) Neuro2a/APPSwe cells showed significant higher NgR gene expression level than Neuro2a cells after 48 h of treatment in 1% FBS medium. (**B**) NgR mRNA level between treatment groups of Neuro2a and Neuro2a/APPSwe cells was normalized vs. 10% FBS group for each cell type. NgR gene expression level in both Neuro2a and Neuro2a/APPSwe cells significantly decreased upon treatment with 10 µg/mL of CM extract for 48 h compared to 1% FBS treatment in each cell type. Values are mean ± SD of at least three independent experiments. * *p*-value < 0.05, ** *p*-value < 0.01.

The gene expression of Nogo receptor (NgR), a downstream signaling protein of Lingo-1, was further investigated. Under a differentiated condition induced by 1% FBS medium control, the APP-overexpressing Neuro2a cells exhibited a significant higher NgR mRNA expression (2.46-fold) than wild-type Neuro2a cells (*p*-value = 0.0029) (Figure 8A). As observed for Lingo-1 gene expression, the NgR mRNA expression was not different between Neuro2a and Neuro2a/APPSwe cells treated with 10% FBS or 1% FBS medium controls, whereas it significantly decreased in both cell lines (*p*-value = 0.0392 and 0.0028, respectively) after exposure to 1% FBS medium plus 10 µg/mL CM extract for 48 h compared to 1% FBS medium control alone (Figure 8B).

### 2.9. Effects of CM Extract on BACE1 Gene Expression

**Figure 9:**
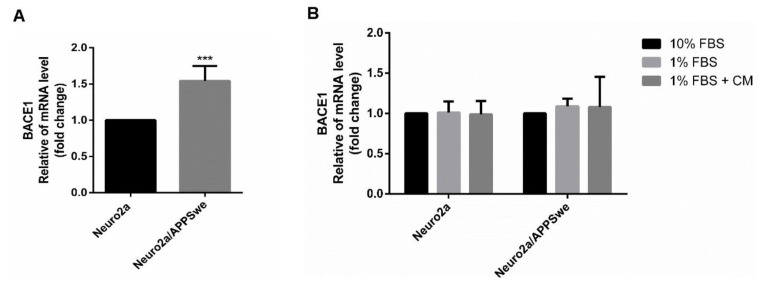
BACE1 mRNA expression level. (**A**) Neuro2a/APPSwe cells showed significantly higher level of BACE1 gene expression than Neuro2a cells after 48 h of treatment in 1% FBS medium. (**B**) BACE1 mRNA level between treatment groups of Neuro2a and Neuro2a/APPSwe cells was normalized vs. 10% FBS group for each cell type. BACE1 gene expression level in both Neuro2a and Neuro2a/APPSwe cells was not altered when treatment with 10 µg/mL of CM extract for 48 h compared to 1% FBS treatment in each cell. Values are mean ± SD of at least three independent experiments. *** *p*-value < 0.001.

When comparing BACE1 gene expression between Neuro2a and Neuro2a/APPSwe cells after incubation in 1% FBS for 48 h, BACE1 mRNA level in Neuro2a/APPSwe cells was significantly higher than in the wild-type counterpart by 1.54 fold (*p*-value = 0.0002) (Figure 9A). Ten percent FBS and 1% FBS treatments elicited no changes in BACE1 gene expression in both Neuro2a and Neuro2a/APPSwe cells after incubation for 48 h. Interestingly, BACE1 mRNA was not influenced by the CM extract at in both wild-type and APP-overexpressing cells (Figure 9B).

### 2.10. Effects of CM Extract on BACE1 Activity

**Figure 10:**
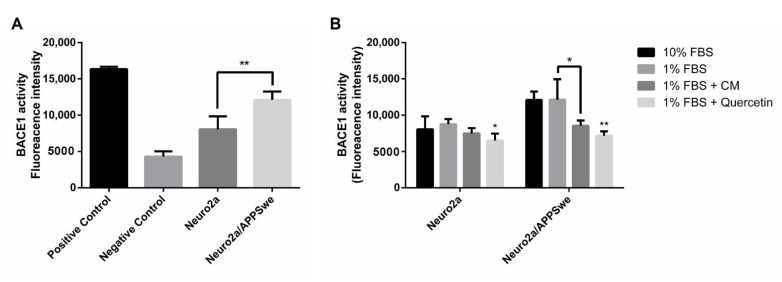
BACE1 activity expressed as fluorescence intensity unit per 30 µg of protein sample. (**A**) Neuro2a/APPSwe cells showed significantly higher level of BACE1 activity than Neuro2a cells after 48 h of treatment in 1% FBS medium. The enzyme activity correlated with the enzyme concentration. (**B**) Quercetin (1 µM), a well-known BACE1 inhibitor, significantly decreased the enzyme activity in both cells. BACE1 activity in Neuro2a/APPSwe cells significantly decreased upon treatment with 10 µg/mL of CM extract for 48 h compared to 1% FBS treatment, and it slightly decreased in Neuro2a cells. Values are mean ± SD of at least three independent experiments. * *p*-value < 0.05, ** *p*-value < 0.01.

BACE1 was assayed according to the kit instructions and the activity was expressed as the fluorescence intensity unit per 30 µg of protein sample. Neuro2a/APPSwe cells showed a significantly higher level of BACE1 activity than Neuro2a cells after 48 h of treatment in 1% FBS medium (Figure 10A). Neuronal cells were treated with CM extract along with quercetin (1 µM), a well-known BACE1 inhibitor, for 48 h. Quercetin treatment significantly decreased the enzyme activity when compared to 1% FBS treatment in each cell type. BACE1 activity in Neuro2a/APPSwe cells was significantly decreased upon treatment with 10 µg/mL of CM extract for 48 h compared to 1% FBS treatment, and it slightly decreased (though not significantly) in Neuro2a cells (Figure 10B).

### 2.11. In Silico Virtual Screening of Binding Affinity between CM-Phytochemical Compounds and BACE1

The current study employed the same batch of the CM methanol extract used in our previous work, where its chemical composition analyzed by LC-MS was reported [33]. Ten most represented CM-phytochemical compounds were tested for their potential to inhibit BACE1 by in silico molecular docking approach (Table 1). For the method validation, 5HA, a reported inhibitor of BACE1 crystal structure, was removed and re-docked into the original active cavity of BACE1 for three runs. The results showed that 5HA was capably re-docked into a similar location and orientation of the original crystal structure with RMSD 1.03, 0.73, and 0.79 Å (less than 2 Å is considered the accuracy for docking [44,45]). Moreover, the predicted binding energies from three analyses were −13.32, −13.06, and −13.19 kcal/mol, demonstrating the acceptable reproducibility of analysis. Protein–ligand interactions exhibited that re-docking conformation of 5HA interacted with key amino acids found in the co-crystallized structure. For example, re-docking run #3, which provided the lowest binding energy, showed that 5HA formed hydrogen bonds with 8 out of 10 amino acids found in co-crystallized complex, including GLY34, SER35, THR72, GLN73, ASP228, GLY230, THR232, and ASN233. Furthermore, it shared amino acid interaction with 6 out of 9 amino acids: LEU30, TYR71, TRP115, THR231, THR232, and ALA335 by hydrophobic bonding (Table 1 and Figure S1). Therefore, these results indicated that the protocol used in this study was reliable and could be applied for further predictions.

**Figure 11:**
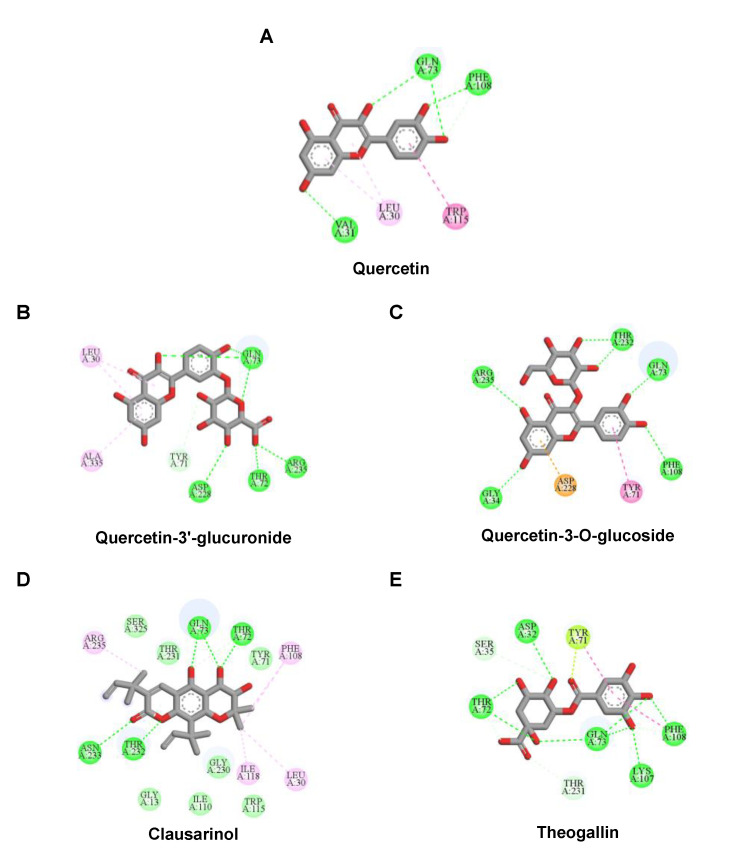
Diagrams represent protein–ligand interactions. Schematics of amino acid interactions of BACE1 and candidate ligands: (**A**) quercetin, (**B**) quercetin-3′-glucuronide, (**C**) quercetin-3-O-glucoside, (**D**) clausarinol, and (**E**) theogallin. Green dashes indicate hydrogen bonds. Pink dashes indicate hydrophobic bonds. Orange dash indicates electrostatic bond.

The molecular docking results of all candidate compounds with BACE1 inhibitory potential were tabulated in Table 2. Quercetin, a natural compound possessing BACE1 inhibitory activity in silico, in vitro, and in vivo [46,47,48], was found in the CM extract (based on HPLC), and it was docked as a reference inhibitor. The binding energy of quercetin with BACE1 was calculated at −8.78 kcal/mol, and this value was set as a reference value for evaluating the ability of inhibitor candidate compounds. Four compounds, including quercetin-3′-glucuronide (−10.74 kcal/mol), quercetin-3-O-glucoside (−10.43 kcal/mol), clausarinol (−9.96 kcal/mol), and theogallin (−8.97 kcal/mol), showed binding energies lower than that of the reference control. These results suggest that quercetin-3′-glucuronide, quercetin-3-O-glucoside, clausarinol, and theogallin have the potential to inhibit BACE1. Diagrams of protein–ligand interactions of these compounds and BACE1 were represented in Figure 11. Schematics of amino acid interactions of BACE1 and CM-phytochemical compounds are shown in Table S1.

### 2.12. ADMET Properties of CM-Phytochemical Compounds

To evaluate the pharmacokinetic and drug-likeness properties, CM-constituent compounds were predicted via pkCSM database [49]. Drug-likeness was indicated by Lipinski’s rule of five and had no more than one violation of the following criteria: molecular weight ≤500, the number of hydrogen bond acceptor ≤10, the number of hydrogen bond donor ≤5, and the Log P_o/w_ ≤ 5 [50]. As shown in Table 3, the predictions found that most compounds passed Lipinski’s rule of five except quercetin-3′-glucuronide and quercetin-3-O-glucoside. Absorption, distribution, metabolism, excretion, and toxicity (ADMET) properties of the compounds are reported in Table 4. Overall, all the CM-phytoconstituents predicted no AMES toxicity. However, bioavailability of these compounds has to be confirmed using in vitro or in vivo models in future experiments.

## 3. Discussion

Several plants and natural products have been studied for use as herbal therapeutics in complementary and alternative medicine [51]. A number of plants and their isolated compounds possessing neurite outgrowth activity have been proposed for the treatment of neurodegenerative diseases. Here, we established an alternative strategy for the discovery of phytochemicals with a potential curative effect in Alzheimer’s disease (AD). Since APP overexpression negatively affects neurite outgrowth activity [12], here, we employed wild-type Neuro2A and APPSwe-overexpressing Neuro2A as an in vitro model to test the neurite outgrowth potential of *C. mimosoides* (CM) extract.

Our previous LC-MS analysis showed that the methanol extract of CM contains the neurite outgrowth stimulatory compounds gallic acid and quercetin [33]. In the present study, HPLC results showed that CM methanol extract exhibited high gallic acid content of 7.8% of the crude extract. On the other hand, only 0.04% quercetin was found. According to our previous report, LC-MS results of CM methanol extract showed a high content of quercetin glycosides, including quercetin-3′-glucuronide and quercetin-3-O-glucoside [33]. It is possible that CM methanol extract mainly contains quercetin glycosides rather than quercetin.

Consistent with the literature [12], we found that neurite outgrowth was inhibited in Swedish mutation APP-overexpressing Neuro2a (Neuro2a/APPSwe) cells when compared to wild-type Neuro2a (Neuro2a/WT) cells after treatment with 1%FBS differentiation medium. Notably, neurite outgrowth activity in both Neuro2a/WT and Neuro2a/APPSwe cells was increased after treatment with CM methanol extract. This study is the first report of neurite outgrowth inducing activity in APP-overexpressing neurons upon treating the cells with CM extract. To give a mechanistic support to this activity, the expression of neurite outgrowth regulating gene was investigated.

The mRNA expression of GAP-43 and Ten-4, two neurite outgrowth positive regulators, was up-regulated in both wild-type and APP-overexpressing neurons after treatment with the CM extract. GAP-43 plays a key role in neurite outgrowth, which is expressed in neuronal growth cones during development [52,53]. It has been reported that the upregulation of GAP-43 by natural products potentiates the neurite outgrowth [54,55,56]. Another neurite outgrowth regulator is Teneurin-4 (Ten-4), a transmembrane protein of the Teneurin family. Ten-4 positively regulates the formation of filopodia-like protrusion and neurite outgrowth [43]. In Neuro2a cells, Ten-4 triggered neurite outgrowth by activation of focal adhesion kinase (FAK) and Rho-family small GTPases, Cdc42 and Rac1, key molecules for the membranous protrusion formation downstream of FAK [39]. Recently, herbal extracts such as *Mucuna pruriens*, *Anacardium occidentale* L., *Glochidion zeylanicum*, *Vitis vinifera*, and *Camellia sinensis* (oolong tea) were also reported to increase neurite outgrowth dependent on Ten-4 expression [31,57,58,59,60].

Lingo-1, or leucine-rich repeat neuronal protein 1, is a transmembrane protein that is highly expressed in the brain, and it is implicated in several neurodegenerative diseases [17,18]. It has been shown that Lingo-1 is capable of directly binding to APP, promoting its proteolytic process via inducing BACE1 cleavage in amyloidogenic pathway and, thus, increasing the production of Aβ fragments [16,18,61]. Its action, resulting in suppression of growth cones and further axonal growth [19], involves the Nogo receptor (NgR) as a part of a co-receptor complex leading to activation of the rho-associated coiled coil-containing protein kinase (RhoA/ROCK) signaling pathway. Several natural products have shown the potential to counteract NgR. For instance, green tea and a variety of Chinese herbal medicines could inhibit the expression of NgR and, consequently, promote neurite outgrowth [62,63,64]. Additionally, the flavonoid isoquercitrin was found to promote neurite elongation via reducing RhoA activity, which is down-stream to the NgR pathway [65].

Here, we show that, compared to the wild-type counterpart, Swedish mutant APP overexpressing neurons exhibit an upregulation of Lingo-1, NgR, and BACE1 gene expression levels, as well as increased BACE1 activity. Remarkably, gene expression levels of Lingo-1 and NgR were suppressed, and neurite outgrowth was induced by CM extract treatment. On the other hand, no alteration was found in the BACE1 gene expression level.

BACE1 activity was then assayed and found decreased in CM extract-treated Neuro2a/APPSwe cells. One micromole per liter of Quercetin, a known BACE1 inhibitor, was employed for reference. Based on the quantification by HPLC, the content of quercetin in 10 µg/mL CM extract used for treatment was 1.2 × 10^−2^ µM. It could be possible that the BACE1 inhibitory effect was due to the mixtures of several bioactive compounds present in the CM extract apart from quercetin. Supporting this notion, the compounds in CM extract with a potential to inhibit BACE1 activity were determined via molecular docking study. Ten CM-phytochemical compounds were docked against the active site of BACE1, and the results found that quercetin-3′-glucuronide, quercetin-3-O-glucoside, clausarinol, and theogallin exhibited more potent activity against BACE1 compared to quercetin, here used as a reference control. Notably, quercetin-3-O-glucoside was previously reported to have a BACE1 inhibitory effect using in vitro non-cell assay by having a half inhibitory concentration (IC_50_) at 41.23 ± 2.31 µM [66]. However, this knowledge about these compounds should be further proven by using higher model systems.

Several plants and natural compounds were reported to possess inhibitory effects against BACE1 activity in APP-overexpressing neuronal cells, among these *Gentiana delavayi* flower extract [67], centipedegrass extract [68], theasaponin E1 from green tea seed [69], and ginsenoside [70]. According to our results, under the CM treatment, APP level and BACE1 gene expression were not decreased, but the activity of this enzyme was decreased. This finding is in agreement with a previous report showing that a quercetin-rich diet in early stages of AD was able to decrease BACE1 activity but did not affect APP and BACE1 mRNA level in APP/PS1 mice model [48,71]. The phytochemicals in CM extract involved in the amyloidogenesis APP cleavage need to be further investigated.

Our ADMET analysis indicates that most of the CM-phytochemical compounds can be absorbed, distributed, metabolized, and excreted well in the human body, along with low toxicity. Therefore, CM extract and its constituent may be suitable as a basis for developing drugs. However, further studies to confirm the oral bioavailability rate and blood–brain barrier penetration of these compounds should be performed.

Taken together, CM extract could counteract (Swe mutant) APP overexpression to stimulate neurite outgrowth through upregulation of GAP-43 and Ten-4 gene expression, along with downregulation of Lingo-1 and NgR gene expression. At least two known neurite outgrowth-inducing compounds, namely, gallic acid and quercetin [36,37,38,39], were found in the CM extract. However, it is likely that other bioactive compounds, yet to be identified, which mainly affect these activities, should be explored, as well as contribute in a synergistic manner to the neurite outgrowth inducing effect of the CM extract. Moreover, we show that the extract is able to inhibit BACE1 activity, possibly due to the presence of quercetin-3′-glucuronide, quercetin-3-O-glucoside, clausarinol, and theogallin.

## 4. Materials and Methods

### 4.1. Materials and Reagents

Gallic acid, quercetin, dimethyl sulfoxide (DMSO), Dulbecco’s modified Eagle’s medium (DMEM), Ham’s F12 medium, and fetal bovine serum (FBS) were purchased from Sigma-Aldrich (St. Louis, MO, USA). Geneticin (G418) was purchased from Invitrogen (San Diego, CA, USA). 3-(4,5-dimethylthiazol-2-yl)-2,5-diphenyltetrazoliumbromide (MTT) was purchased from Bio Basic (Markham, Ontario, Canada). Trizol reagent was purchased from Invitrogen (Carlsbad, CA, USA). Phosphate buffer saline (PBS) was HyClone^TM^ and purchased from GE Healthcare Bio-Sciences, USA. EmbryoMax^®^ non-essential amino acids (NEAA) solution was purchased from Millipore^®^ (Burlington, MA, USA). Penicillin/Streptomycin solution was purchased from Gibco (Waltham, MA, USA). Cell lysis buffer (10X), anti-APP, anti-β-actin, and horseradish peroxidase-coupled secondary antibody were purchased from Cell Signaling Technology (Danvers, MA, USA). Methanol was purchased from Merck (Darmstadt, Germany).

### 4.2. Cell Culture

Mouse neuroblastoma cells used in this study include wild-type APP expressing Neuro2a (Neuro2a) cells and Swedish mutant APP-overexpressing Neuro2a (Neuro2a/APPSwe) cells. Neuro2a cells were obtained from the Health Science Research Resources Bank (Osaka, Japan). Neuro2a/APPSwe cells were kindly provided by Professor Ciro Isidoro [40,41]. Both cell lines were maintained in DMEM and Ham’s F12 medium (ratio 1:1) supplemented with 10% FBS, 1% NEAA, and 1% penicillin/streptomycin. A low concentration of G418 (0.4%) was added to the culture medium for Neuro2a/APPSwe cells to maintain transgene expression. The cells were grown in a humidified incubator with 5% CO_2_ at 37 °C.

### 4.3. Plant Extract Preparation

The CM methanol extract was from the same batch of extract that was used in our previous study [33]. In brief, twigs and leaves of *C. mimosoides* (CM) were collected from the local market in Chiang Rai Province, Thailand. The plant was authenticated and deposited with voucher specimen number A014170 (BCU) at the herbarium of Kasin Suvatabhandhu (Department of Botany, Faculty of Science, Chulalongkorn University, Bangkok, Thailand). The twigs and leaves were dried and crushed into a powder. Then, approximately 40 g of the dried powder was constantly packed into a thimble and extracted in a Soxhlet apparatus with 400 mL of methanol for 24 h. The appearing supernatant was collected, filtrated, and evaporated to dryness under vacuum. The yield of CM methanol extract was 29.82% (*w*/*w*). To stabilize the chemical composition in the extract, the stock of dried crude extract was kept at −20 °C and protected from light exposure. Finally, the extract was prepared as a stock solution of 100 mg/mL in DMSO, sterilized through a 0.2 µm pore size syringe filter, stored at −20 °C, and protected from light until further use.

### 4.4. HPLC Analysis

The amount of gallic acid and quercetin were quantified using high-performance liquid chromatography (HPLC) analysis. The assay was performed at RSU Science and Technology Research Equipment Center (Rangsit University, Pathum Thani, Thailand). Gallic acid and quercetin reference compounds were accurately weighed and freshly prepared in 0.05 M perchloric acid containing 0.1 mM Na_2_EDTA on ice and stored at −20 °C prior to use. SHIMADZU LC-10 HPLC, equipped with an analytical C18 reversed-phase column (ODS3 C18, 4.6 × 250 mm i.d., 5-micrometer particle size), and UV detector were used. The mobile phase consisted of 0.02 M sodium acetate, buffered to a pH of 4 with 0.0125 M citric acid, containing 0.042 M methanesulfonic acid and 0.1 mM EDTA, and the flow rate was 1 mL/min. The calibration curves were prepared by injecting a series of gallic acid and quercetin standard dilutions. Gallic acid and quercetin in CM methanol extract were quantified by means of calibration curves obtained from the standards.

### 4.5. MTT Assay

The cells were cultured in a 96-well plate at a density of 5000 cells per well and incubated for 24 h. Then, the cells were treated with different concentrations (1, 5, 10, 25, 50, and 100 µg/mL) of CM extract. After 48 h of incubation, the medium was removed and MTT solution was added to the cells at a final concentration of 0.5 mg/mL. After an additional incubation for 3 h, the solution was carefully removed, and the formazan crystal was solubilized in 150 μL DMSO. The optical density (OD) was measured using an EnSpire^®^ Multimode Plate Reader (Perkin-Elmer, Waltham, MA, USA) at 550 nm.

### 4.6. Neurite Outgrowth Assay

The cells were seeded in a 6-well plate at an initial density of 5000 cells per well in 2 mL 10% FBS medium and incubated for 24 h. Then, the medium was carefully removed, and the cells were washed by PBS prior to treating with differentiation medium (1% FBS medium) with the CM extract for another 48 h. The extract was prepared by diluting the stock solution in 1% FBS medium at the selected optimum concentration that showed cell viability by more than 90%. Tests with the cells cultured in 10% FBS and 1% FBS medium were also conducted in parallel as negative controls. After completion of incubation period, the cells were visualized under 10× magnification using the differential interphase contrast (DIC) microscope (Axio Observer A1, Carl Zeiss, Köln, Germany) at 10× magnification, photographed using a Canon EOS50D camera, and processed with ImageJ Software (National Institutes of Health, Bethesda, MD, USA). For identification of neurite-bearing cells, a cell was scored positive if it bore a thin neurite extension that was double or more the length of the cell body diameter. For neurite length determination, the longest length of neurite of the cell was measured from cell membrane of cell body to the end of growth cone. At least 100 cells per well in 10–20 random microscopic fields were examined to calculate the percentage of neurite-bearing cells and the average length of neurite per cell.

### 4.7. RNA Extraction and Reverse Transcription-Polymerase Chain Reaction (RT-PCR)

The cells were seeded in a 6-well plate at an initial density of 10,000 cells per well in 2 mL of 10% FBS medium and incubated for 24 h. After carefully removing the medium, the cells were washed with PBS and subsequently treated with differentiation medium (1% FBS medium) with the selected optimal concentration of CM extract for another 48 h. Experiments with the cells cultured in 10% FBS and 1% FBS medium were also conducted in parallel as negative controls. Total RNA was extracted from the cells using Trizol reagent following the manufacturer’s instructions. The amount of RNA was measured by absorbance at 260 nm using NanoDrop™ 1000 Spectrophotometer (Thermo Fisher Scientific, Fitchburg, WI, USA). One microgram of total RNA was used for cDNA synthesis using AccuPower RT PreMix (Bioneer Co., Daejeon, Korea) and Oligo(dT) 17 primer.

### 4.8. Quantitative Real-Time PCR Analysis

Real-time PCR analysis was used to determine the mRNA expression level. The reaction was performed in the Exicycler^TM^ version 3.0 (Bioneer Co., Daejeon, Korea). The amplification was done using Greenstar^TM^ qPCR Premix (Bioneer Co., Daejeon, Korea) and the primers listed in Table 5. The thermal cycling condition was composed of an initial denaturation step at 95 °C for 10 min, followed by 40 cycles of 95 °C for 15 s, appropriate annealing temperature for 15 s, and 72 °C for 30 s. The relative change in gene expression was analyzed using the 2^−ΔΔCt^ method, where the data of cycle threshold (C_t_) values of each target gene were normalized to that of β-actin for controlling the variability in expression levels. Each treatment was run in at least triplicate.

### 4.9. Western Blot Analysis

The cells were seeded overnight in a 6-well plate at an initial density of 10,000 cells per well in 10% FBS medium and incubated for 24 h. The medium was removed, and the cells were washed by PBS and subsequently treated with differentiation medium (1% FBS medium) with the selected optimal concentration of CM extract for another 48 h. Tests on the cells cultured in 10% FBS and 1% FBS medium were also conducted in parallel as negative controls. After completion of incubation period, cell lysates were prepared in 1X cell lysis buffer and quantified for total protein concentrations using Bradford assay. An equal amount of protein (15 μg) from each treatment was first denatured by heating in Laemmli loading buffer at 95 °C for 10 min and then separated on 10% SDS polyacrylamide gel prior transfer to PVDF membrane. Following blocking for 1 h with 5% skim milk in TBS-T (Tris-buffered saline with 0.1% Tween 20), the membranes were allowed to incubate overnight at 4 °C with primary antibodies specific for APP (1:5000) or β-actin (1:5000) and further probed with HRP-conjugated secondary antibodies (1:10,000) at room temperature for 60 min. The immune complexes were visualized using the enhanced chemiluminescence method (ECL™ Select Western blotting detection reagent: GE Healthcare, Piscataway, NJ, USA). Images of protein bands were captured by the DCP-165C brother scanner and evaluated using ImageJ software (National Institutes of Health). The expression level of APP was calculated using β-actin as the normalization control.

### 4.10. Determination of BACE1 Activity

The cells were seeded overnight at density of 10,000 cells per well in a 6-well plate and subsequently cultured and treated as mentioned above. Additionally, the cells treated with quercetin (1 µM), a well-known BACE1 inhibitor, were also determined. After that, cell lysates were prepared in 1X cell lysis buffer and quantified for total protein concentrations using Bradford assay. An equal amount of protein (30 μg) from each condition was investigated for BACE1 activity using BACE1 activity Fluorometric Assay Kit (Sigma-Aldrich, St. Louis, MO, USA). An active BACE1 solution and a mixing of active BACE1 solution and BACE1 inhibitor provided by the kit were used as positive and negative controls, respectively.

### 4.11. In Silico Virtual Screening of Binding Affinity between Candidate Ligands and BACE1

#### 4.11.1. Protein Preparation

Crystal structure of BACE1 complexed with 5HA (N’-[(2S,3R)-4-(cyclopropylamino)-3-hydroxy-1-phenyl-butan-2-yl]-5-(methyl-methylsulfonyl-amino)-N-[(1R)-1-phenylethyl]benzene-1,3-dicarboxamide) inhibitor (PDB ID: 3TPP) was retrieved from RCSB Protein Data Bank [73]. The protein was prepared by using AutoDockTools (version 1.5.6) software. All water molecules and the original ligand presented in the complex structure were deleted. All missing hydrogens and Kollman charges were added to the protein structure. Then, the format file was generated in pdbqt format and set for further study.

#### 4.11.2. Ligand Preparation

The structures of all compounds were generated from the SMILES strings by using BIOVIA Draw 2019. Clean geometry and generating of pdb format of all compounds were performed using BIOVIA Discovery Studio Visualizer (version 20.1). The format file of ligands was converted to pdbqt format using AutoDockTools 1.5.6.

#### 4.11.3. Molecular Docking

Molecular docking study was adapted from the previous report [74]. In brief, the grid box was set based on the original ligand at the active site of BACE1 with the number of points in xyz-dimension of 35 × 35 × 35, spacing at 0.375 Å, and center grid box at 29.959 × 41.005 × 3.292 (xyz). The rigid docking was performed by AutoDock 4.2 with Lamarckian GA algorithm and default parameters. Ten conformations of the protein–ligand complex were generated; the conformation that provided the lowest binding energy was reported.

#### 4.11.4. Protein–Ligand Interaction

The interactions between BACE1 and candidate ligands were visualized by the BIOVIA Discovery Studio Visualizer (version 20.1). The hydrogen bonds, hydrophobic bonds, and electrostatic bonds of amino acid interactions were considered.

### 4.12. ADMET Analysis

The structures of CM phytochemical compounds (Table 2) were obtained from PubChem database in SMILE files for evaluating the pharmacokinetic properties. ADMET were predicted via pkCSM database [49].

### 4.13. Statistical Analysis

All experiments were carried out in at least three independent experiments. The experimental data were analyzed using GraphPad Prism version 6 (GraphPad Software Inc., San Diego, CA, USA) and expressed as mean ± standard deviation (SD). Statistical differences between two groups were analyzed by independent *t*-test. The comparison of more than two groups was analyzed by one-way analysis of variance (ANOVA), followed by Tukey’s post-hoc test. The *p*-value < 0.05 was considered to be significant.

## 5. Conclusions

This study shows that CM methanol extract possesses neurite outgrowth properties. This effect was observed not only in the wild-type neurons but also in Swedish APP-over-expressing neuronal cells, indicating that CM extract counteracted APP interference in the neurite outgrowth. The known neurite outgrowth-inducing compounds, gallic acid and quercetin, were found in the CM extract. Additionally, other bioactive compounds yet to be identified in the CM extract likely contributed synergistically to this effect. The possible mechanisms include the upregulation of the neurite outgrowth activation genes GAP-43 and Ten-4 and the downregulation of the neurite outgrowth negative regulator genes NgR and Lingo-1. Moreover, BACE1 activity was inhibited after treatment with CM extract. The potential CM phytochemical compounds responsible for BACE1 inhibition indicated by the molecular docking study are quercetin-3′-glucuronide, quercetin-3-O-glucoside, clausarinol, and theogallin. Notably, all the major CM-phytoconstituents were predicted not to be toxic. From these data, we conclude that CM twigs and leaves could be a potential natural source for developing novel therapeutic drugs for AD. Additional studies will better clarify the active constituents responsible for promoting neurite outgrowth and BACE1 inhibition and the molecular mechanisms underlying such activities. In the future, we shall test in animal experimental AD models the beneficial effects of these compounds in view of the translation into clinics.

## Figures and Tables

**Table 1:** The results of method validation between co-crystalized ligand (5HA) at the original active site of BACE1 (PDB ID: 3TPP).

Ligand	RMSD (Å)	Binding Energy (kcal/mol)	Amino Acid Interaction		
			Hydrogen Bond	Hydrophobic Bond	Electrostatic Bond
5HA (crystal structure) 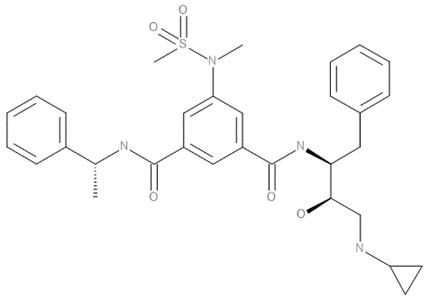	Not determined	Not determined	ASP32 GLY34 SER35 THR72 (2) GLN73 (2) ASP228 GLY230 (2) THR231 THR232 (2) ASN233	LEU30 TYR71 ILE110 TRP115 (2) TYR198 ILE226 THR231 (2) THR232 (2) ALA335	-
5HA (re-docking run #1)	1.03	−13.32	ASP32 THR72 (3) GLN73 (2) ASP228 GLY230 THR231 THR232	GLN12 GLY13 TYR71 THR231 THR232 ALA335	-
5HA (re-docking run #2)	0.73	−13.60	ASP32 GLY34 THR72 (2) GLN73 (2) GLY230 THR232 ASN233 (2) SER325	LEU30 TYR71 TRP115 TYR198 ILE226 THR231 THR232 VAL332 ALA335	-
5HA (re-docking run #3)	0.79	−13.94	GLY34 SER35 THR72 GLN73 (2) ASP228 GLY230 (2) THR232 ASN233 ARG235 SER325	LEU30 TYR71 PHE108 TRP115 (2) THR231 (2) THR232 ALA335	-

**Table 2:** The results of molecular docking between the active site of BACE1 (PDB ID: 2WJO) and candidate ligands.

Ligand	Binding Energy (kcal/mol)	Inhibition Constant	Amino Acid Interaction		
			Hydrogen Bond	Hydrophobic Bond	Electrostatic Bond
Quercetin (reference inhibitor) 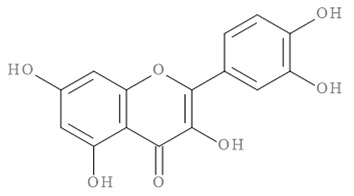	−8.78	365.72 µM	VAL31 GLN73 (2) PHE108 (2)	LEU30 (2) TRP115	-
3-O-Methylgallate 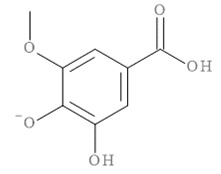	−5.20	154.27 µM	THR72 GLN73 (3) PHE108 THR232 (2)	PHE108	-
4-Aminomethylindole 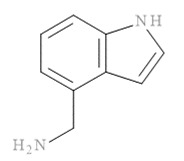	−6.49	17.63 µM	-	TYR71 (2) GLY230 THR231	-
Bergenin 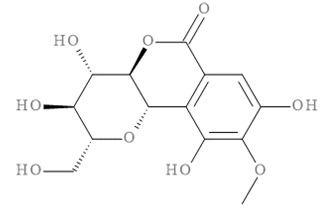	−8.52	572.8 nM	TYR71 THR72 GLN73 THR232	-	-
Clausarinol 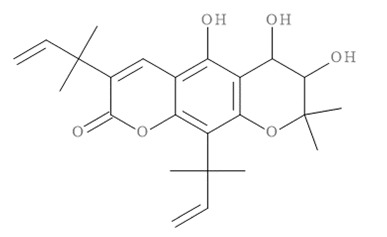	−9.96	50.27 nM	THR72 (2) GLN73 (2) THR232 (2) ASN233	LEU30 PHE108 (2) ILE118 ARG235	-
Emmotin A 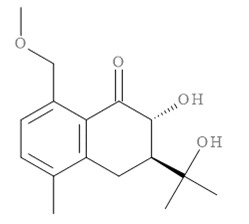	−7.90	1.62 µM	THR72 (3) THR232 THR231	TYR71 PHE108 ILE118	-
Gallic acid 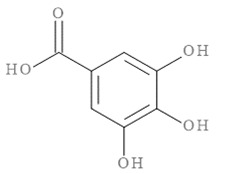	−5.09	186.3 µM	THR72 GLN73 (2) THR232 (2)	-	-
N-D-Glucosylarylamine 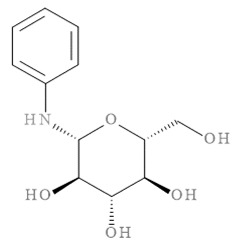	−7.10	6.29 µM	GLN73 (2) PHE108 GLY230 THR231 (2) THR232 (2)	-	ASP228
Quercetin-3′-glucuronide 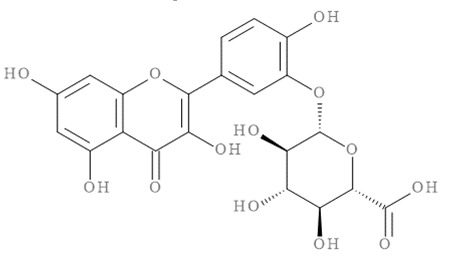	−10.74	13.45 nM	TYR71 THR72 GLN73 (3) ASP228 ARG235 (2)	LEU30 (2) ALA335	-
Quercetin-3-O-glucoside 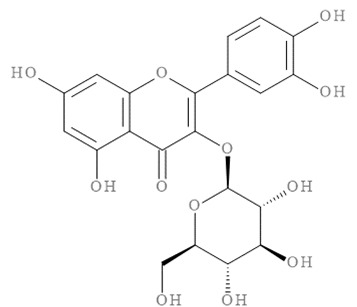	−10.43	22.54 nM	GLY34 GLN73 PHE108 (2) THR232 (3) ARG235	ASP228	TYR71
Theogallin 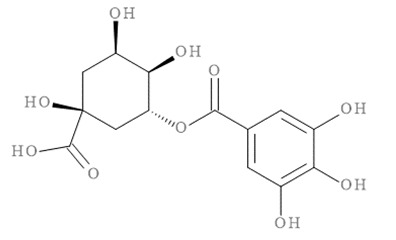	−8.97	266.1 nM	THR72 (3) GLN73 (3) ASP32 SER35 LYS107 PHE108 (2) THR231	TYR71 PHE108	-

**Table 3:** The physiochemical properties of CM-phytoconstituents based on Lipinski’s rule of five.

Compound	MW (g/mol) (≤500 g/mol)	Num. H-Bond Acceptors (≤10)	Num. H-Bond Donors (≤5)	Log P_o/w_ (≤5)	Violation (≤1)
3-O-Methylgallate	183.139	4	2	0.1726	0
4-Aminomethylindole	146.193	1	2	1.6266	0
Bergenin	328.273	9	5	-1.2006	0
Clausarinol	414.498	6	3	3.9911	0
Emmotin A	278.348	4	2	1.62822	0
Gallic acid	170.120	4	4	0.5016	0
N-D-Glucosylarylamine	255.270	6	5	−1.1016	0
Quercetin	302.238	7	5	1.988	0
Quercetin-3′-glucuronide	478.362	12	8	−0.4466	2
Quercetin-3-O-glucoside	464.379	12	8	−0.5389	2
Theogallin	344.272	9	7	−1.3399	1

**Table 4:** ADMET analysis of CM-phytoconstituents.

Compound	Water Solubility (log mol/L)	Intestinal Absorption (% Absorbed)	BBB Permeability (log BB)	CNS Permeability (log PS)	CYP2D6 Substrate	CYP2D6 Inhibitor	Total Clearance (log mL/min/kg)	AMES Toxicity	Max. Tolerated Dose in Human (log mg/kg/day)
3-O-Methylgallate	−2.094	73.513	−0.198	−2.807	No	No	0.694	No	1.169
4-Aminomethylindole	−1.847	91.463	0.352	−2.091	No	No	0.991	No	−0.323
Bergenin	−1.853	63.774	−1.091	−3.903	No	No	0.427	No	−0.013
Clausarinol	−3.641	79.297	−0.982	−2.136	No	No	0.394	No	−0.151
Emmotin A	−2.886	95.021	−0.113	−2.903	No	No	1.051	No	0.536
Gallic acid	−2.560	43.374	−1.102	−1.102	No	No	0.518	No	0.700
N-D-Glucosylarylamine	−1.628	42.217	−0.671	−3.486	No	No	0.217	No	0.788
Quercetin	−2.925	77.207	−1.098	−3.065	No	No	0.407	No	0.499
Quercetin-3′-glucuronide	−2.894	0.999	−1.656	−4.117	No	No	0.429	No	0.432
Quercetin-3-O-glucoside	−2.925	47.999	−1.688	−4.093	No	No	0.394	No	0.569
Theogallin	−2.589	22.529	−1.679	−4.166	No	No	0.594	No	0.246

BBB: blood–brain barrier, BB: brain:blood drug concentration ratio, CNS: central nervous system, PS: permeability–surface area.

**Table 5:** Primer sequences, product sizes, and annealing temperatures used for quantitative real-time PCR.

Primer	Sequence (Forward and Reverse)	Product Size (bp)	Annealing Temperature (°C)
GAP-43	5′- AGCCTAAACAAGCCGATGTG -3′ 5′- GGTTTGGCTTCGTCTACAGC -3′	157	62
Ten-4 [43]	5′- GTGGACAAGTTTGGGCTCATTTA -3′ 5′- GGGTTGATGGCTAAGTCTGTGG -3′	185	62
Lingo-1 [72]	5′- TCTATCACGCACTGCAACCTGAC -3′ 5′- AGCATGGAGCCCTCGATTGTA -3′	116	56
NgR	5′- CCTGCAGAGGTCCTAATGCC -3′ 5′- GAGGCGCTTAAGATCACGGT -3′	180	60
BACE1	5′- CCAGGGCTACTATGTGGAGATGA -3′ 5′- GTGTCCACCAGGATGTTGAGC -3′	66	58
β-actin	5′- GGCTGTATTCCCCTCCATCG -3′ 5′- CCAGTTGGTAACAATGCCATGT -3′	154	62

## Data Availability

The data used to support the findings of this study are included within the article and supplementary information file.

## Supplementary Materials

The following are available online at https://www.mdpi.com/article/10.3390/ph14090901/s1, Figure S1: Diagrams represent protein-ligand interactions. Table S1: Diagrams of protein-ligand interactions of BACE1 and candidate ligands.

## References

[1] Murphy M.P., LeVine H. (2010). Alzheimer’s disease and the amyloid-beta peptide. J. Alzheimer’s Dis..

[2] De-Paula V.J., Radanovic M., Diniz B.S., Forlenza O.V. (2012). Alzheimer’s disease. Sub-Cell. Biochem..

[3] Hamano T., Gendron T.F., Causevic E., Yen S.H., Lin W.L., Isidoro C., Deture M., Ko L.W. (2008). Autophagic-lysosomal perturbation enhances tau aggregation in transfectants with induced wild-type tau expression. Eur. J. Neurosci..

[4] Tiraboschi P., Hansen L.A., Thal L.J., Corey-Bloom J. (2004). The importance of neuritic plaques and tangles to the development and evolution of AD. Neurology.

[5] Serrano-Pozo A., Frosch M.P., Masliah E., Hyman B.T. (2011). Neuropathological alterations in Alzheimer disease. Cold Spring Harb. Perspect. Med..

[6] Gong C.X., Iqbal K. (2008). Hyperphosphorylation of microtubule-associated protein tau: A promising therapeutic target for Alzheimer disease. Curr. Med. Chem..

[7] Prasansuklab A., Tencomnao T. (2013). Amyloidosis in Alzheimer’s Disease: The Toxicity of Amyloid Beta (A beta), Mechanisms of Its Accumulation and Implications of Medicinal Plants for Therapy. Evid.-Based Complementary Altern. Med..

[8] Luu L., Ciccotosto G.D., Vella L.J., Cheng L., Roisman L.C., Multhaup G., Hill A.F., Munter L.M., Cappai R. (2019). Amyloid Precursor Protein Dimerisation Reduces Neurite Outgrowth. Mol. Neurobiol..

[9] Thinakaran G., Koo E.H. (2008). Amyloid precursor protein trafficking, processing, and function. J. Biol. Chem..

[10] Mullan M., Houlden H., Windelspecht M., Fidani L., Lombardi C., Diaz P., Rossor M., Crook R., Hardy J., Duff K. (1992). A locus for familial early-onset Alzheimer’s disease on the long arm of chromosome 14, proximal to the alpha 1-antichymotrypsin gene. Nat. Genet..

[11] Scheuner D., Eckman C., Jensen M., Song X., Citron M., Suzuki N., Bird T.D., Hardy J., Hutton M., Kukull W. (1996). Secreted amyloid beta-protein similar to that in the senile plaques of Alzheimer’s disease is increased in vivo by the presenilin 1 and 2 and APP mutations linked to familial Alzheimer’s disease. Nat. Med..

[12] Wang Y.P., Wang Z.F., Zhang Y.C., Tian Q., Wang J.Z. (2004). Effect of amyloid peptides on serum withdrawal-induced cell differentiation and cell viability. Cell Res..

[13] Kiryushko D., Berezin V., Bock E. (2004). Regulators of neurite outgrowth: Role of cell adhesion molecules. Ann. N. Y. Acad. Sci..

[14] Read D.E., Gorman A.M. (2009). Involvement of Akt in neurite outgrowth. Cell. Mol. Life Sci..

[15] Li H.L., Roch J.M., Sundsmo M., Otero D., Sisodia S., Thomas R., Saitoh T. (1997). Defective neurite extension is caused by a mutation in amyloid beta/A4 (A beta) protein precursor found in familial Alzheimer’s disease. J. Neurobiol..

[16] Bai Y., Markham K., Chen F., Weerasekera R., Watts J., Horne P., Wakutani Y., Bagshaw R., Mathews P.M., Fraser P.E. (2008). The in vivo brain interactome of the amyloid precursor protein. Mol. Cell. Proteom..

[17] Andrews J.L., Fernandez-Enright F. (2015). A decade from discovery to therapy: Lingo-1, the dark horse in neurological and psychiatric disorders. Neurosci. Biobehav. Rev..

[18] Fernandez-Enright F., Andrews J.L. (2016). Lingo-1: A novel target in therapy for Alzheimer’s disease?. Neural Regen. Res..

[19] Shao Z., Browning J.L., Lee X., Scott M.L., Shulga-Morskaya S., Allaire N., Thill G., Levesque M., Sah D., McCoy J.M. (2005). TAJ/TROY, an orphan TNF receptor family member, binds Nogo-66 receptor 1 and regulates axonal regeneration. Neuron.

[20] Wang K.C., Kim J.A., Sivasankaran R., Segal R., He Z. (2002). P75 interacts with the Nogo receptor as a co-receptor for Nogo, MAG and OMgp. Nature.

[21] Calabrese E.J. (2008). Enhancing and regulating neurite outgrowth. Crit. Rev. Toxicol..

[22] Huang E.J., Reichardt L.F. (2001). Neurotrophins: Roles in neuronal development and function. Annu. Rev. Neurosci..

[23] Larkfors L., Ebendal T., Whittemore S.R., Persson H., Hoffer B., Olson L. (1987). Decreased level of nerve growth factor (NGF) and its messenger RNA in the aged rat brain. Brain Res..

[24] Bartus R.T. (2000). On neurodegenerative diseases, models, and treatment strategies: Lessons learned and lessons forgotten a generation following the cholinergic hypothesis. Exp. Neurol..

[25] Budni J., Bellettini-Santos T., Mina F., Garcez M.L., Zugno A.I. (2015). The involvement of BDNF, NGF and GDNF in aging and Alzheimer’s disease. Aging Dis..

[26] Granholm A.C., Albeck D., Backman C., Curtis M., Ebendal T., Friden P., Henry M., Hoffer B., Kordower J., Rose G.M. (1998). A non-invasive system for delivering neural growth factors across the blood-brain barrier: A review. Rev. Neurosci..

[27] Pardridge W.M. (2002). Neurotrophins, neuroprotection and the blood-brain barrier. Curr. Opin. Investig. Drugs.

[28] Nitta A., Murakami Y., Furukawa Y., Kawatsura W., Hayashi K., Yamada K., Hasegawa T., Nabeshima T. (1994). Oral administration of idebenone induces nerve growth factor in the brain and improves learning and memory in basal forebrain-lesioned rats. Naunyn-Schmiedeberg’s Arch. Pharmacol..

[29] Tohda C., Kuboyama T., Komatsu K. (2005). Search for natural products related to regeneration of the neuronal network. Neuro-Signals.

[30] More S.V., Koppula S., Kim I.S., Kumar H., Kim B.W., Choi D.K. (2012). The role of bioactive compounds on the promotion of neurite outgrowth. Molecules.

[31] Duangjan C., Rangsinth P., Zhang S., Wink M., Tencomnao T. (2021). *Anacardium Occidentale*, L. Leaf Extracts Protect Against Glutamate/H_2_O_2_-Induced Oxidative Toxicity and Induce Neurite Outgrowth: The Involvement of SIRT1/Nrf2 Signaling Pathway and Teneurin 4 Transmembrane Protein. Front. Pharmacol..

[32] Chanwitheesuk A., Teerawutgulrag A., Rakariyatham N. (2005). Screening of antioxidant activity and antioxidant compounds of some edible plants of Thailand. Food Chem..

[33] Rangsinth P., Prasansuklab A., Duangjan C., Gu X., Meemon K., Wink M., Tencomnao T. (2019). Leaf extract of *Caesalpinia mimosoides* enhances oxidative stress resistance and prolongs lifespan in Caenorhabditis elegans. BMC Complementary Altern. Med..

[34] Yodsaoue O., Karalai C., Ponglimanont C., Tewtrakul S., Chantrapromma S. (2010). Potential anti-inflammatory diterpenoids from the roots of *Caesalpinia mimosoides* Lamk. Phytochemistry.

[35] Rattanata N., Klaynongsruang S., Daduang S., Tavichakorntrakool R., Limpaiboon T., Lekphrom R., Boonsiri P., Daduang J. (2016). Inhibitory Effects of Gallic Acid Isolated from *Caesalpinia mimosoides* Lamk on Cholangiocarcinoma Cell Lines and Foodborne Pathogenic Bacteria. Asian Pac. J. Cancer Prev..

[36] Tangsaengvit N., Kitphati W., Tadtong S., Bunyapraphatsara N., Nukoolkarn V. (2013). Neurite Outgrowth and Neuroprotective Effects of Quercetin from *Caesalpinia mimosoides* Lamk. on Cultured P19-Derived Neurons. Evid.-Based Complementary Altern. Med..

[37] Pervin M., Unno K., Ohishi T., Tanabe H., Miyoshi N., Nakamura Y. (2018). Beneficial Effects of Green Tea Catechins on Neurodegenerative Diseases. Molecules.

[38] Nakajima K.-I., Niisato N., Marunaka Y. (2011). Quercetin stimulates NGF-induced neurite outgrowth in PC12 cells via activation of Na^+^/K^+^/2Cl^−^ cotransporter. Cell. Physiol. Biochem..

[39] Chen M.-M., Yin Z.-Q., Zhang L.-Y., Liao H. (2015). Quercetin promotes neurite growth through enhancing intracellular cAMP level and GAP-43 expression. Chin. J. Nat. Med..

[40] Cagnin M., Ozzano M., Bellio N., Fiorentino I., Follo C., Isidoro C. (2012). Dopamine induces apoptosis in APPswe-expressing Neuro2A cells following Pepstatin-sensitive proteolysis of APP in acid compartments. Brain Res..

[41] Resende R., Ferreira-Marques M., Moreira P., Coimbra J.R.M., Baptista S.J., Isidoro C., Salvador J.A.R., Dinis T.C.P., Pereira C.F., Santos A.E. (2020). New BACE1 Chimeric Peptide Inhibitors Selectively Prevent AβPP-β Cleavage Decreasing Amyloid-β Production and Accumulation in Alzheimer’s Disease Models. J. Alzheimer’s Dis..

[42] Shigeta M., Shibukawa Y., Ihara H., Miyoshi E., Taniguchi N., Gu J. (2006). Beta1,4-N-Acetylglucosaminyltransferase III potentiates beta1 integrin-mediated neuritogenesis induced by serum deprivation in Neuro2a cells. Glycobiology.

[43] Suzuki N., Numakawa T., Chou J., de Vega S., Mizuniwa C., Sekimoto K., Adachi N., Kunugi H., Arikawa-Hirasawa E., Yamada Y. (2014). Teneurin-4 promotes cellular protrusion formation and neurite outgrowth through focal adhesion kinase signaling. FASEB J..

[44] Gohlke H., Hendlich M., Klebe G. (2000). Knowledge-based scoring function to predict protein-ligand interactions. J. Mol. Biol..

[45] Ramírez D., Caballero J. (2018). Is It Reliable to Take the Molecular Docking Top Scoring Position as the Best Solution without Considering Available Structural Data?. Molecules.

[46] Shimmyo Y., Kihara T., Akaike A., Niidome T., Sugimoto H. (2008). Flavonols and flavones as BACE-1 inhibitors: Structure–activity relationship in cell-free, cell-based and in silico studies reveal novel pharmacophore features. Biochim. Biophys. Acta (BBA)-Gen. Subj..

[47] Mphahlele M.J., Agbo E.N., More G.K., Gildenhuys S. (2021). In Vitro Enzymatic and Kinetic Studies, and In Silico Drug-Receptor Interactions, and Drug-Like Profiling of the 5-Styrylbenzamide Derivatives as Potential Cholinesterase and β-Secretase Inhibitors with Antioxidant Properties. Antioxidants.

[48] Lu Y., Liu Q., Yu Q. (2018). Quercetin enrich diet during the early-middle not middle-late stage of alzheimer’s disease ameliorates cognitive dysfunction. Am. J. Transl. Res..

[49] Pires D.E.V., Blundell T.L., Ascher D.B. (2015). pkCSM: Predicting Small-Molecule Pharmacokinetic and Toxicity Properties Using Graph-Based Signatures. J. Med. Chem..

[50] Lipinski C.A., Lombardo F., Dominy B.W., Feeney P.J. (2001). Experimental and computational approaches to estimate solubility and permeability in drug discovery and development settings. Adv. Drug Deliv. Rev..

[51] Schachter S.C. (2009). Botanicals and herbs: A traditional approach to treating epilepsy. Neurotherapeutics.

[52] Benowitz L.I., Routtenberg A. (1997). GAP-43: An intrinsic determinant of neuronal development and plasticity. Trends Neurosci..

[53] Aarts L.H., Schotman P., Verhaagen J., Schrama L.H., Gispen W.H. (1998). The role of the neural growth associated protein B-50/GAP-43 in morphogenesis. Adv. Exp. Med. Biol..

[54] Gundimeda U., McNeill T.H., Schiffman J.E., Hinton D.R., Gopalakrishna R. (2010). Green tea polyphenols potentiate the action of nerve growth factor to induce neuritogenesis: Possible role of reactive oxygen species. J. Neurosci. Res..

[55] Lai H.C., Wu M.J., Chen P.Y., Sheu T.T., Chiu S.P., Lin M.H., Ho C.T., Yen J.H. (2011). Neurotrophic effect of citrus 5-hydroxy-3,6,7,8,3′,4′-hexamethoxyflavone: Promotion of neurite outgrowth via cAMP/PKA/CREB pathway in PC12 cells. PLoS ONE.

[56] Phan C.W., David P., Wong K.H., Naidu M., Sabaratnam V. (2015). Uridine from *Pleurotus giganteus* and Its Neurite Outgrowth Stimulatory Effects with Underlying Mechanism. PLoS ONE.

[57] Wansawat S., Mani Iyer P., Isidoro C., Tencomnao T. (2018). *Mucuna pruriens* Seed Extract Promotes Neurite Outgrowth via TEN-4 Dependent and Independent Mechanisms in NEURO2A Cells. Sains Malays..

[58] Duangjan C., Rangsinth P., Zhang S., Gu X., Wink M., Tencomnao T. (2021). Neuroprotective Effects of *Glochidion zeylanicum* Leaf Extract against H_2_O_2_/Glutamate-Induced Toxicity in Cultured Neuronal Cells and Aβ-Induced Toxicity in *Caenorhabditis elegans*. Biology.

[59] Duangjan C., Rangsinth P., Zhang S., Gu X., Wink M., Tencomnao T. (2021). *Vitis Vinifera* Leaf Extract Protects Against Glutamate-Induced Oxidative Toxicity in HT22 Hippocampal Neuronal Cells and Increases Stress Resistance Properties in *Caenorhabditis Elegans*. Front. Nutr..

[60] Zhang S., Duangjan C., Tencomnao T., Liu J., Lin J., Wink M.J.F. (2020). Function, Neuroprotective effects of oolong tea extracts against glutamate-induced toxicity in cultured neuronal cells and β-amyloid-induced toxicity in *Caenorhabditis elegans*. Food Funct..

[61] de Laat R., Meabon J.S., Wiley J.C., Hudson M.P., Montine T.J., Bothwell M. (2015). LINGO-1 promotes lysosomal degradation of amyloid-β protein precursor. Pathobiol. Aging Age Relat. Dis..

[62] Fan T.K., Gundimeda U., Mack W.J., Gopalakrishna R. (2016). Counteraction of Nogo-A and axonal growth inhibitors by green tea polyphenols and other natural products. Neural Regen. Res..

[63] Liu W., Zhou Y., Jia Q., Han B., Zhang G. (2011). Effects of Fujian tablet on Nogo-A mRNA expression and plasticity of the corticospinal tract in a rat model of focal cerebral ischemia. Neural Regen. Res..

[64] Qin X.D., Kang L.Y., Liu Y., Huang Y., Wang S., Zhu J.Q. (2012). Chinese Medicine’s Intervention Effect on Nogo-A/NgR. Evid.-Based Complementary Altern. Med..

[65] Palazzolo G., Horvath P., Zenobi-Wong M. (2012). The flavonoid isoquercitrin promotes neurite elongation by reducing RhoA activity. PLoS ONE.

[66] Jung H.A., Karki S., Kim J.H., Choi J.S. (2015). BACE1 and cholinesterase inhibitory activities of Nelumbo nucifera embryos. Arch. Pharmacal Res..

[67] Yang M., Zhou K.Y., Li F.F., Yang H.Y., Yin M., Zhang L.H., Wang F.S. (2020). Effects of *Gentiana delavayi* Flower Extract on APP Processing in APP/PS1 CHO Cells. Biol. Pharm. Bull..

[68] Song Y., Kim H.D., Lee M.K., Kim M.K., Kang S.N., Ko Y.G., Won C.K., Kim G.S., Lee S.S., Bai H.W. (2015). Protective effect of centipedegrass against Aβ oligomerization and Aβ-mediated cell death in PC12 cells. Pharm. Biol..

[69] Khan M.I., Shin J.H., Kim M.Y., Shin T.S., Kim J.D. (2020). Green Tea Seed Isolated Theasaponin E1 Ameliorates AD Promoting Neurotoxic Pathogenesis by Attenuating Aβ Peptide Levels in SweAPP N2a Cells. Molecules.

[70] Cao G., Su P., Zhang S., Guo L., Zhang H., Liang Y., Qin C., Zhang W. (2016). Ginsenoside Re reduces Aβ production by activating PPARγ to inhibit BACE1 in N2a/APP695 cells. Eur. J. Pharmacol..

[71] Di Meo F., Valentino A., Petillo O., Peluso G., Filosa S., Crispi S. (2020). Bioactive Polyphenols and Neuromodulation: Molecular Mechanisms in Neurodegeneration. Int. J. Mol. Sci..

[72] Wang C.J., Qu C.Q., Zhang J., Fu P.C., Guo S.G., Tang R.H. (2014). Lingo-1 inhibited by RNA interference promotes functional recovery of experimental autoimmune encephalomyelitis. Anat. Rec..

[73] Xu Y., Li M.J., Greenblatt H., Chen W., Paz A., Dym O., Peleg Y., Chen T., Shen X., He J. (2012). Flexibility of the flap in the active site of BACE1 as revealed by crystal structures and molecular dynamics simulations. Acta Crystallogr. Sect. D Biol. Crystallogr..

[74] Rangsinth P., Sillapachaiyaporn C., Nilkhet S., Tencomnao T., Ung A.T., Chuchawankul S. (2021). Mushroom-derived bioactive compounds potentially serve as the inhibitors of SARS-CoV-2 main protease: An in silico approach. J. Tradit. Complementary Med..

