# Supplementary material for: Caesalpinia mimosoides Leaf Extract Promotes Neurite Outgrowth and Inhibits BACE1 Activity in Mutant APP-Overexpressing Neuronal Neuro2a Cells
Source: Pharmaceuticals (Basel). 2021 Sep 4;14(9):901. doi: 10.3390/ph14090901 (PMC8469274; doi:10.3390/ph14090901)
Supplement: Supplementary file 1 [file pharmaceuticals-14-00901-s001.zip › pharmaceuticals-1339263-supplementary.pdf]

Supplementary Materials

Caesalpinia mimosoides Leaf Extract Promotes Neurite Outgrowth and Inhibits BACE1 Activity in Mutant APP-Overexpressing Neuronal Neuro2a Cells

Panthakarn Rangsinth, Chatrawee Duangjan, Chanin Sillapachaiyaporn, Ciro Isidoro,

Anchalee Prasansuklab and Tewin Tencomnao

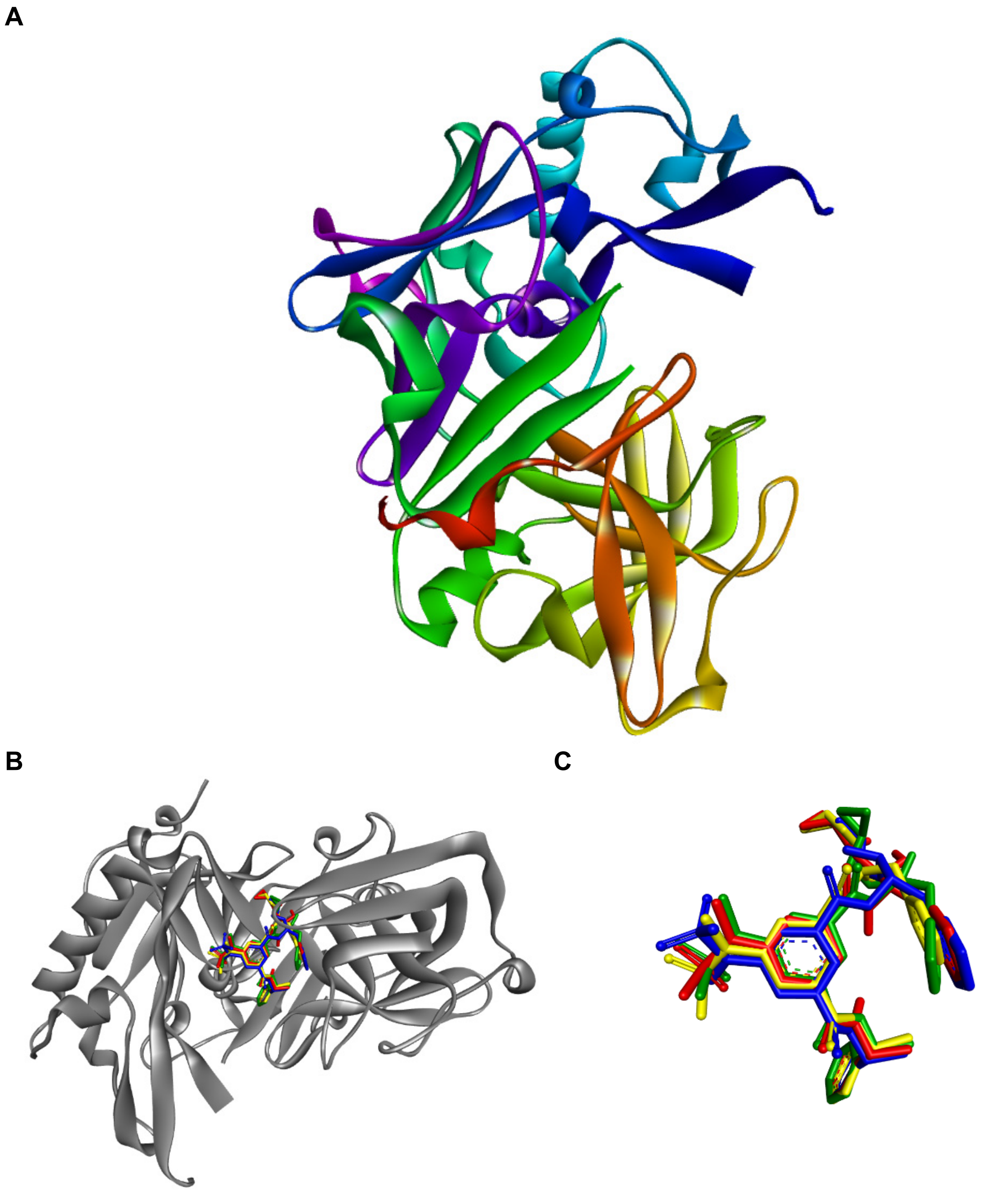

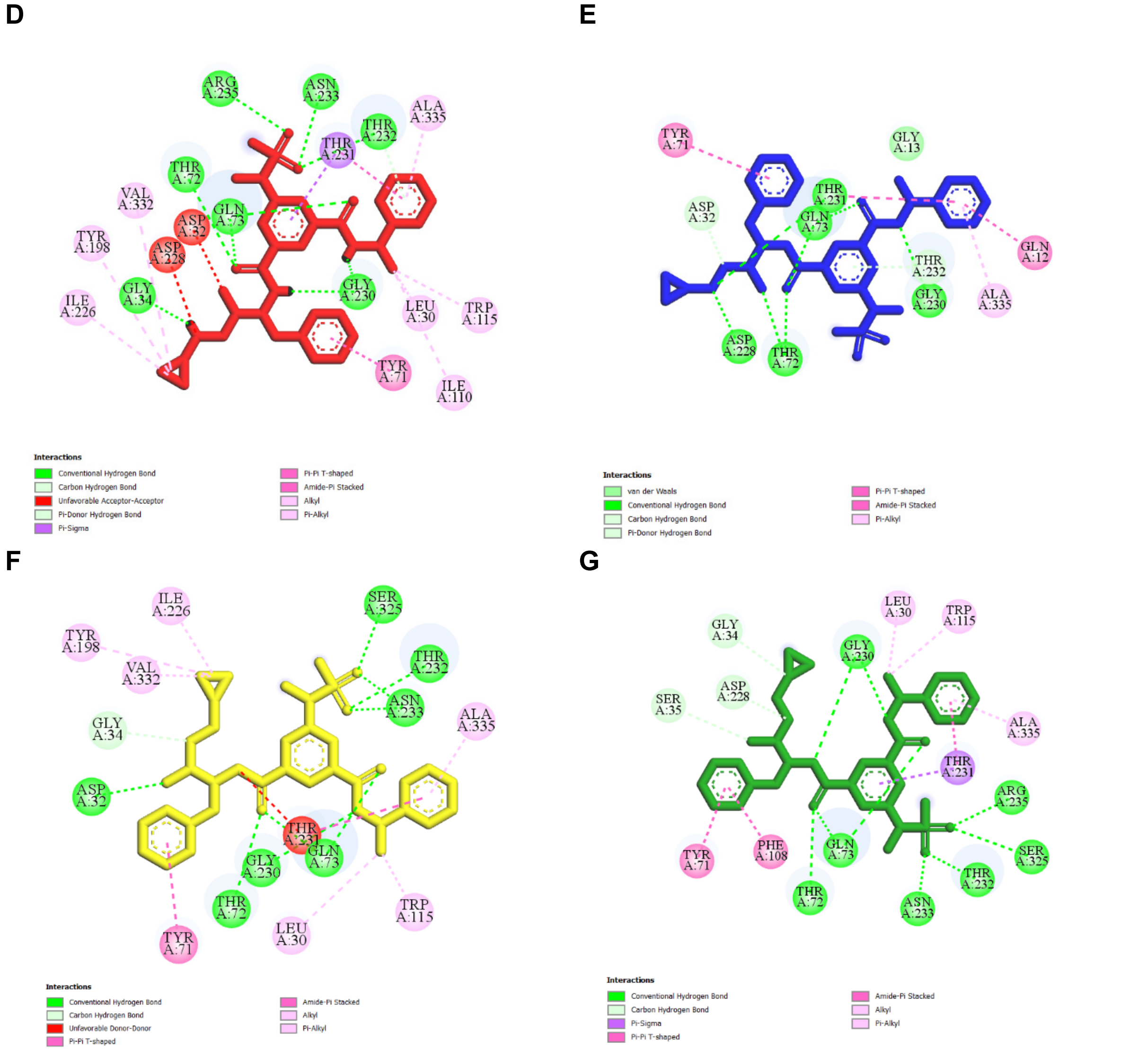

Figure S1. Diagrams represent protein-ligand interactions. (A) BACE1 structure (PBB ID 3TPP) (B-C) Overlapping structures of 5HA original crystal structure (red) and re-docking conformation on BACE1 (blue, yellow, and green are from redocking run 1-3, respectively. Schematics of amino acid interactions of BACE1 and 5HA: (D) 5HA from crystal structure, (E) 5HA from re-docking run 1, (F) 5HA from re-docking run 2, and (G) 5HA from re-docking run 3.

Table S1. Diagrams of protein-ligand interactions of BACE1 and candidate ligands.

<table>
<tr><th>Ligand</th><th colspan="2">Protein-ligand interactions</th></tr>
<tr><td>Quercetin
(reference inhibitor)</td><td>

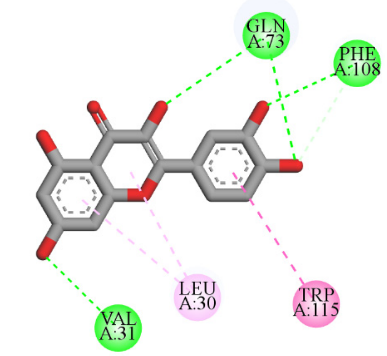

</td><td>

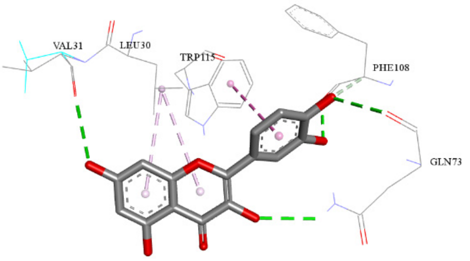

</td></tr>
<tr><td>3-O-Methylgallate</td><td>

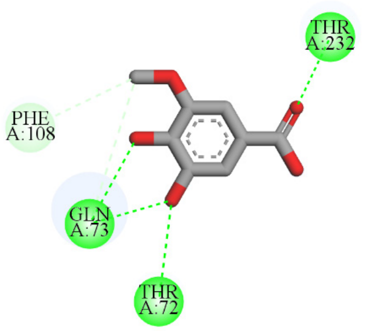

</td><td>

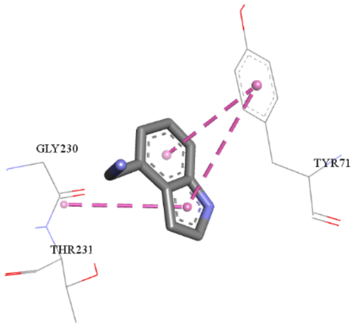

</td></tr>
<tr><td>4-Aminomethylindole</td><td>

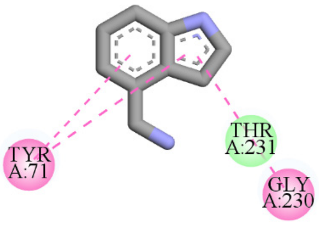

</td><td>

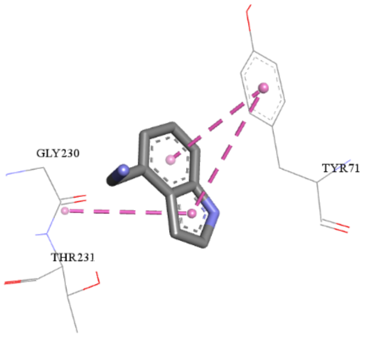

</td></tr>
<tr><td>Bergenin</td><td>

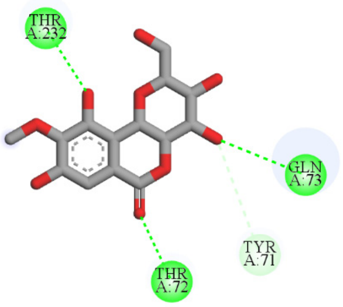

</td><td>

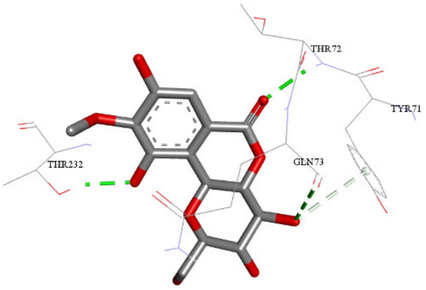

</td></tr>
</table>

Ligand	Protein-ligand interactions	
Clausarinol	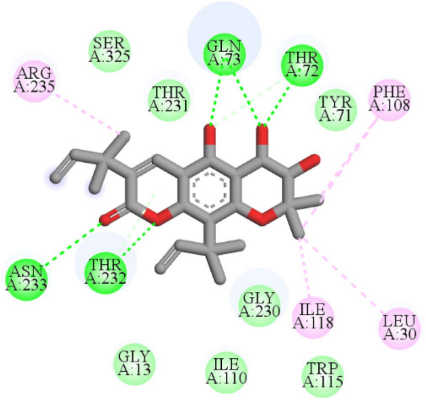	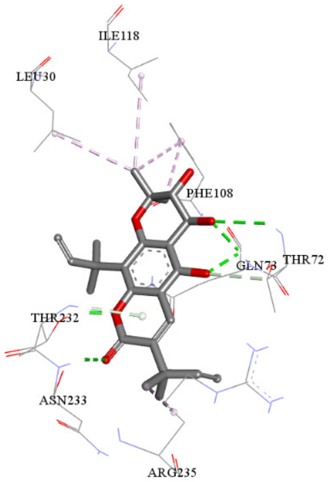
Emmotin A	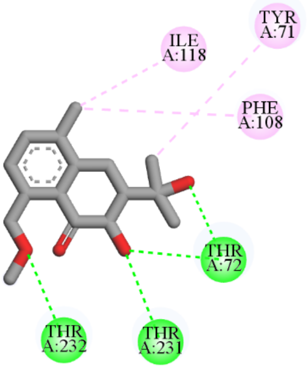	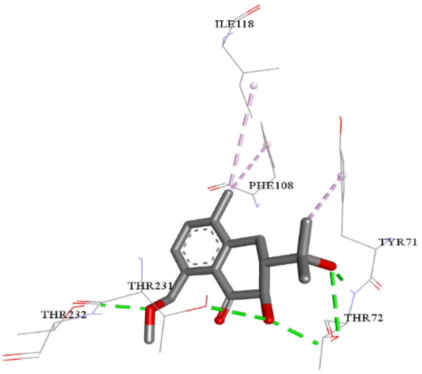
Gallic acid	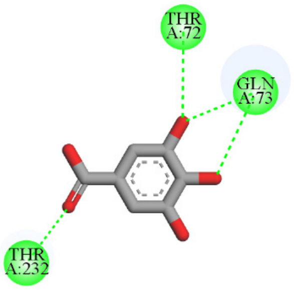	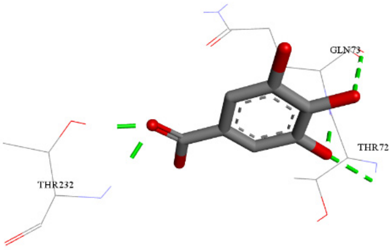

<table>
<tr><th>Ligand</th><th colspan="2">Protein-ligand interactions</th></tr>
<tr><td>N-D-Glucosylarylamine</td><td>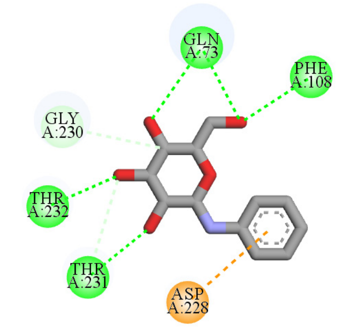
</td><td>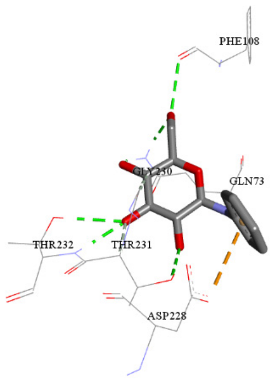
</td></tr>
<tr><td>Quercetin-3'-glucuronide</td><td>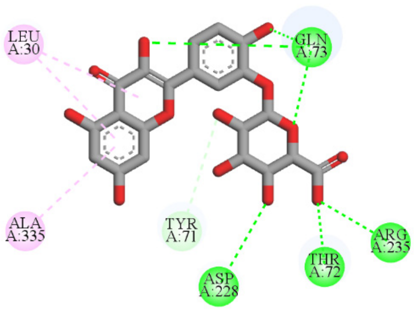
</td><td>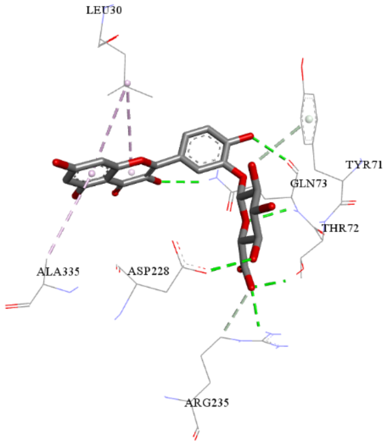
</td></tr>
<tr><td>Quercetin-3-O-glucoside</td><td>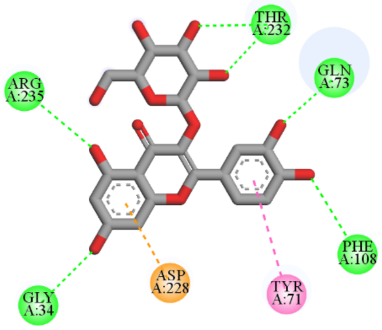
</td><td>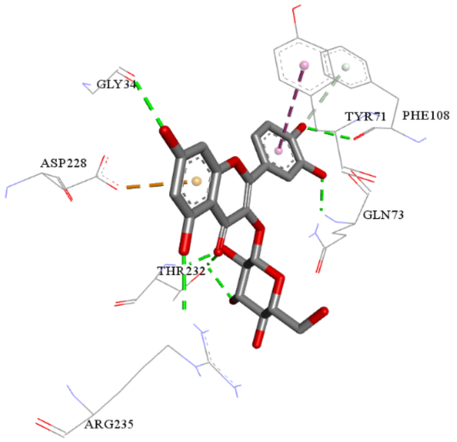
</td></tr>
</table>

Ligand	Protein-ligand interactions	
Theogallin	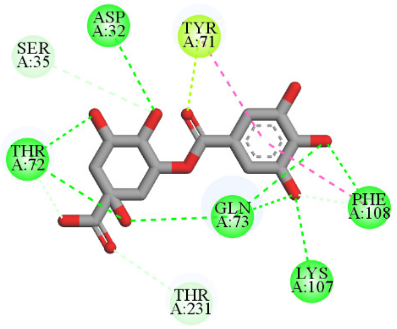	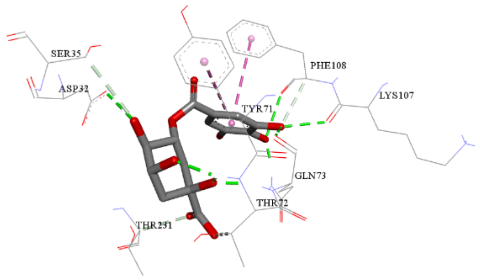

Green-dashes indicate hydrogen bonds. Pink-dashes indicate hydrophobic bonds. Orange-dashes indicate electrostatic bonds.
